# MicroRNA profiling of low concentration extracellular vesicle RNA utilizing NanoString nCounter technology

**DOI:** 10.1002/jex2.72

**Published:** 2023-01-28

**Authors:** Rachel E. Crossland, Anna Albiero, Clara Sanjurjo‐Rodríguez, Monica Reis, Anastasia Resteu, Amy E. Anderson, Anne M. Dickinson, Arthur G. Pratt, Mark Birch, Andrew W. McCaskie, Elena Jones, Xiao‐nong Wang

**Affiliations:** ^1^ Translational and Clinical Research Institute, Faculty of Medical Sciences Newcastle University Newcastle upon Tyne UK; ^2^ Division of Trauma and Orthopaedic Surgery, Department of Surgery University of Cambridge Addenbrooke's Hospital Cambridge UK; ^3^ Physiotherapy, Medicine and Biomedical Sciences department, University of A Coruña; University Hospital Complex from A Coruña (Sergas, CHUAC Institute of Biomedical Research of A Coruña (INIBIC)‐Centre of Advanced Scientific Researches (CICA) A Coruña Spain; ^4^ Centre for Regenerative Medicine, Institute for Regeneration and Repair The University of Edinburgh, Edinburgh BioQuarter Edinburgh UK; ^5^ Musculoskeletal Services Directorate Newcastle upon Tyne Hospitals NHS Foundation Trust UK; ^6^ Leeds Institute of Rheumatic and Musculoskeletal Medicine University of Leeds Leeds UK

**Keywords:** extracellular vesicle, microRNA, NanoString, profiling

## Abstract

Extracellular vesicles (EV) and the microRNAs that they contain are increasingly recognised as a rich source of informative biomarkers, reflecting pathological processes and fundamental biological pathways and responses. Their presence in biofluids makes them particularly attractive for biomarker identification. However, a frequent caveat in relation to clinical studies is low abundance of EV RNA content. In this study, we used NanoString nCounter technology to assess the microRNA profiles of *n* = 64 EV low concentration RNA samples (180–49125 pg), isolated from serum and cell culture media using precipitation reagent or sequential ultracentrifugation. Data was subjected to robust quality control parameters based on three levels of limit of detection stringency, and differential microRNA expression analysis was performed between biological subgroups. We report that RNA concentrations > 100 times lower than the current NanoString recommendations can be successfully profiled using nCounter microRNA assays, demonstrating acceptable output ranges for imaging parameters, binding density, positive/negative controls, ligation controls and normalisation quality control. Furthermore, despite low levels of input RNA, high‐level differential expression analysis between biological subgroups identified microRNAs of biological relevance. Our results demonstrate that NanoString nCounter technology offers a sensitive approach for the detection and profiling of low abundance EV‐derived microRNA, and may provide a solution for research studies that focus on limited sample material.

## 1 INTRODUCTION

First described over 35 years ago, extracellular vesicles (EVs) are an important class of lipid bilayer particles that are secreted in the extracellular space by nearly all prokaryotic and eukaryotic cells (Yáñez‐Mó et al., 2015). The EV family includes a spectrum of sub‐categories, including shedding vesicles that derive from the plasma membrane (such as microvesicles and apoptotic bodies: 150–1000 nm), as well as smaller vesicles (such as exosomes: 30–100 nm), which are generated in the endosomal compartment by inward budding of multivesicular bodies (MVB) and released upon fusion of MVB with the plasma membrane (Colombo et al., 2014). Such sub‐categories can be broadly distinguished by their origin, size distribution, molecular content and physiological role (Van Niel et al., 2018).

Initially thought to contain unwanted cellular ‘rubbish’, compelling evidence has since shown that EVs represent important mediators of autocrine, paracrine and endocrine signalling pathways, allowing for intercellular transmission of macromolecules between cells (Iraci et al., 2016). Indeed, EVs have been implicated to play a fundamental role in a broad range of biological functions and pathological conditions. As such, there has been an explosion of basic and translational research within the field, towards establishing a complete understanding of their involvement in both health and disease (Iraci et al., 2016).

Extracellular vesicles boast a rich molecular composition, and comprehensive characterisation studies have led to the establishment of various databases to publicly document their content (Kalra et al., 2012; Kim et al., 2015). Their molecular makeup includes surface receptors, membrane and soluble proteins, lipids, genomic DNA, and a heterogeneous range of RNA including mRNA, microRNA, tRNA, rRNA, small nucleolar RNA, small circular nucleolar RNA, piRNA, scaRNA, viral RNA, Y RNA, and long noncoding RNA (Bellingham et al., 2012; Gezer et al., 2014; Huang et al., 2013; Kogure et al., 2013; Takahashi et al., 2014; Vojtech et al., 2014). As the specific content of isolated EVs can reflect biological events and disease processes, comprehensively understanding their EV molecular profiles is key to elucidating their full roles.

Further interest in EVs developed from the finding that they can be detected in a variety of biological fluids (e.g., blood, urine, saliva, amniotic fluid, malignant ascites, bronchoalveolar lavage fluid, synovial fluid and breast milk) (Raposo & Stoorvogel, 2013), where they protect their nucleic acid content from degradation. They can therefore serve as a rich source of minimally‐invasive and informative biomarkers, reflecting pathological processes. This is particularly exciting in relation to microRNAs, which are abundantly present in EVs and are functionally transferred to target cells, where they are able to silence target genes (Ismail et al., 2013; Montecalvo et al., 2012; Pegtel et al., 2010; Valadi et al., 2007). This unique source of diagnostic, prognostic and predictive microRNA biomarkers  has the potential to refine clinical decisions and influence personalised therapy, as well as lead to novel target identification and the development of new therapeutics.

Current widely accepted methods to study the microRNA profiles of EVs include qRT‐PCR, high throughput sequencing and microRNA microarrays. Each has their advantages and disadvantages in relation to throughput, sensitivity, ability to detect novel microRNAs, requirement for bioinformatic interpretation and cost of application, which have been previously reviewed (Godoy et al., 2019; Pritchard et al., 2012). A more recent innovation in microRNA profiling is the nCounter platform from NanoString Technologies. This hybridization‐based system uses two sequence‐specific capture probes that are targeted to microRNAs of interest, allowing multiplexed detection of multiple microRNAs in a single reaction, without the need for amplification or cloning. This ‘molecular barcoding’ approach allows for similar microRNA variants to be discriminated with high accuracy. The NanoString human microRNA panels include over 800 mature targets, based on microRNAs that have been sequenced with high confidence, and/or found to be clinically relevant, covering > 92% of the most abundant microRNAs in miRBase v22 ( NanoString Technologies, 2019). However, in many experimental conditions related to EVs, it can be challenging to reach the standard RNA minimum input requirements specified by NanoString of 100 ng. This is especially the case in serum and plasma biofluid EV studies, where the volume of starting material may be limited by clinical sample availability. However, this 100 ng recommendation is based on miRNA profiling of tissues and cells, and makes the assumption that the RNA profile is similar to that observed in most tissues, whereby 90% of the RNA is ribosomal, 9%–10% is mRNA and less than 1% is small RNA (NanoString Technologies, 2019). Although information on the microRNA composition of EVs is variable (Turchinovich et al., 2019), studies performed in human plasma, saliva and urine indicate that they contain a significant proportion of microRNA reads (35%–76%) (Cheng et al., 2014; Huang et al., 2013; Ogawa et al., 2013; Turchinovich et al., 2019) and thus, NanoString RNA input load can potentially be lowered to reflect this altered composition. This would enable research studies restricted by very limited quantities of RNA.

In this study, we report that nCounter microRNA panels can be successfully applied to assess the microRNA profiles of 64 EV samples with isolated input RNA concentrations up to 100 times lower than that of current standard NanoString recommendations. These included EVs isolated from a variety of sources including serum and cell culture supernatants, using both ultracentrifugation and precipitation reagent methods. Output data was subjected to robust quality control parameters and high‐level profiling analysis of biological sub‐groups within each data set, to demonstrate biologically relevant results.

## 2 MATERIALS AND METHODS

### 2.1 Extracellular vesicle isolation

Extracellular vesicles isolated from serum or cell conditioned medium by precipitation were prepared from 1 to 3 ml of serum or 2 ml of tissue culture supernatant using Total Exosome Isolation Reagent from serum or from cell culture media (ThermoFisher), as appropriate, according to the supplier's protocols. Resultant EV pellets were processed immediately for EV characterisation, or downstream RNA isolation. Preparation of EVs from cell conditioned medium by differential ultracentrifugation (UC) was carried out according to a previously described centrifugation and UC protocol (Reis et al., 2018; Théry et al., 2006). Briefly, conditioned medium was centrifuged at 400 × g for 5 min at 4°C to exclude detached cells and debris, followed by 2000 × g for 20 min at 4°C. Supernatant was transferred to UC tubes (Beckman Coulter) and centrifuged at 10,000 × g for 45 min, followed by at 100,000 × g for 90 min at 4°C using a 45Ti rotor (Beckman Coulter) in a Beckman L8‐80 ultracentrifuge (Beckman Coulter). The EV pellet was washed in 60 ml of PBS, centrifuged again at 100,000 × g for 90 min at 4°C, then re‐suspended in > 100 μl of sterile particle free PBS (Gibco) and stored at −80°C for downstream EV characterisation (Théry et al., 2018) or RNA isolation.

### 2.2 Extracellular vesicle characterisation

#### 2.2.1 Transmission electron microscopy

Transmission electron microscopy (TEM) was performed using 300‐mesh grids (Gilder Grids) filmed with Pioloform® (SPI Supplies), that were carbon coated and plasma etched before use. Each EV pellet was re‐suspended in 100 μl phosphate‐buffered saline (PBS) and a 10 μl droplet picked up by each grid, incubated for 5 s and excess sample removed. The grid was stained with uranyl acetate (Agar Scientific), air‐dried and examined using a Hitachi HT7800 transmission electron microscope. Digital images were collected using an Emsis Xarosa camera with Radius software, in conjunction with the Electron Microscopy Research Services, Newcastle University.

#### 2.2.2 Nanoparticle tracking analysis

Nanoparticle tracking analysis (NTA) was performed using a NanoSight LM10‐HS microscope (Malvern Panalytical Ltd) equipped with NTA software v2.3 (NanoSight Ltd). Background extraction was applied and the automatic setting for minimum expected particle size, minimum track length and blur settings were employed. Three 60 s recordings were recorded for each sample, which was diluted at 1:10,000 in sterile filtered PBS (Sigma). Only measurements with > 1000 completed tracks were analysed.

#### 2.2.3 Flow cytometry

For flow cytometric (FC) assessment of EV surface markers CD64, CD9 and CD81, EVs were coated onto 4 μm aldehyde/sulphate latex beads (ThermoFisher) by overnight incubation at room temperature, using a rotary wheel. Free reactive sites were then blocked with 1 M Glycine (Sigma) for 30 min at room temperature. The beads were washed three times in a PBS + 0.5% Foetal Calf Serum (FCS) solution (Invitrogen), incubated for 2 h at 4°C with anti‐human PE CD63 (H5C6), PerCPCy5.5 CD9 (M‐L13) and APC CD81 (JS‐81) antibodies or corresponding isotype controls (all from BD Biosciences) and then washed a final time. Data acquisition was performed using a FACS Canto II cytometer (BD Biosciences) and analysed with FlowJo v10.0 software (Tree Star Inc., USA).

#### 2.2.4 Western blot

Western blot (WB) for Alix and Flotillin‐1 was performed by lysis of EVs using 2% sodium dodecyl sulphate (SDS) and manual shearing (1 ml syringe and 0.8 × 38 mm of 21G needle (Terumo)). Protein quantification was determined using the Micro BCA™ Protein Assay Kit (ThermoFisher) following manufacturer's directions. Absorbance values were measured using a Microplate Reader (Thermo Labsystems Multiskan Ascent 354) with 575 nm wavelength. Protein lysates were diluted in Laemmli buffer containing 0.2% bromophenol (Fisher Scientific) and 50 μl/ml of β‐merceptoethanol (Sigma) and heated at 95°C prior to loading onto a 4%–20% Mini‐PROTEAN® TGX™ Precast Gel (Bio‐Rad Laboratories) alongside controls and molecular Precision Plus Protein™ Dual Colour Standards (Bio‐Rad Laboratories). Transfer onto PVDF membranes was achieved by electroblotting. Blots were blocked with agitation and blocking buffer (PBS + 0.1% Tween 20, 5% skimmed milk and 5% BSA). Blots were incubated with 1:1000 primary antibody and 5 ml PBS‐Tween 20 (PBS‐T) with 5% skimmed milk and 0.5% BSA. Secondary antibodies were applied using 1:1500 (Polyclonal Goat anti‐Mouse, Daco) and PBS‐T. Blots were visualized under chemiluminescence detection using clarity reagent (Bio‐Rad), the LI‐COR Odyssey FC Imaging System and Image Studio software (LI‐COR).

### 2.3 RNA isolation and concentration

Total RNA was isolated from EVs using the Total Exosome and Protein Isolation Kit (Invitrogen), as per the manufacturer's instructions. RNA was concentrated to ∼25 μl using a NanoString Technologies‐approved centrifugation protocol, incorporating Amicon Ultra‐0.5 Centrifugal Filter Units (Merck Millipore) (Technologies, 2019). All RNA was quantified using the Bioanalyzer (Agilent) and RNA 6000 Pico kit (Agilent).

### 2.4 NanoString nCounter MicroRNA analysis

Total RNA was profiled using the nCounter^®^ Human v3 miRNA Expression Assay Kit (NanoString Technologies). The codeset incorporated 827 human microRNAs and included six positive controls, eight negative controls, six ligation controls, and five mRNA reference controls (*ACTB, B2M, GAPDH, RPL19* and *RPLP0*). Input material comprised 5 μl of concentrated RNA. Data normalization was performed using nSolver Analysis Software v4.0 (NanoString Technologies) which can be freely downloaded and updated at: https://nanostring.com/products/analysis‐solutions/ncounter‐analysis‐solutions/ and is compatible with both Macintosh (10.10–10.11) and Windows (8.1 and 10) operating systems, based on R3.3.2 and XQuartz to run nSolver for Mac. Online resources including video tutorials, manuals and related documents are freely available. Positive control normalisation was performed using the geometric mean and normalisation flagging outside the normalisation factor range 0.3–3.0. Codeset content normalisation was performed using all microRNAs for normalisation above the LowLOD, MedLOD or HighLOD threshold, based on geometric mean and flagging outside the normalisation factor range 0.1–10.0. Thresholds were defined as: Low LOD = average of negative controls; Medium LOD = average of negative controls + 2*Standard Deviation (S.D); High LOD = 2*(average of negative controls + 2*S.D). Background signal was removed for each analysis by manual removal of all microRNAs with counts below each threshold LOD. Fold change (FC) expression differences were calculated using nSolver ratio data, based on normalized count data. Further analysis was performed using a pipeline designed by Newcastle University, Haematological Sciences Department, as previously described (Crossland et al., 2017). Briefly, this integrated a number of “R” (R project) statistical packages in the “R” programming language. Heatmaps were constructed using “gplots” (v3.1.0) and “RColorBrewer” (v1.1‐2), based on an unsupervised clustering approach of the normalized expression counts, with a Euclidean (L2 norm) distance measure and “Complete” as the agglomeration method.

## 3 RESULTS

### 3.1 Sample data sets

Data A comprised of *n* = 12 EV samples isolated by precipitation reagent (PR) from the serum of patients attending an early arthritis clinic. Four biological replicates for each of three diagnostic categories were analysed; *n* = 4 were diagnosed with rheumatoid arthritis (RA) (Group 1), *n* = 4 were diagnosed with osteoarthritis (OA) (Group 2) and *n* = 4 were diagnosed with ‘other inflammatory arthritis’ (OIA) (Group 3). All patients were naïve to immune modulating therapy. Data B comprised of *n* = 36 EV samples isolated from the serum of patients who had undergone haematopoietic stem cell transplantation. All EVs were isolated by PR. Of the *n* = 36 patients, *n* = 24 subsequently developed a systemic complication of transplantation (graft vs. host disease, GvHD) (Group 2), while *n* = 12 did not (Group 1). Data C included *n* = 8 EV samples isolated by PR from the cell culture supernatant of mesenchymal stromal cell (MSC) and cartilage explant (matrix and chondrocyte) co‐cultures, taken from two different regions of knee joints. This included *n* = 4 from joint area 1 (Group 1) and *n* = 4 from joint area 2 (Group 2). Finally, Data D comprised of *n* = 8 EV samples derived from the cell culture supernatant of primary MSCs and isolated by differential ultracentrifugation (UC). This included *n* = 4 samples from healthy donors (Group 1) and *n* = 4 samples from patients suffering from osteoarthritis (Group 2).

### 3.2 Extracellular vesicle and RNA characterisation

**FIGURE 1:**
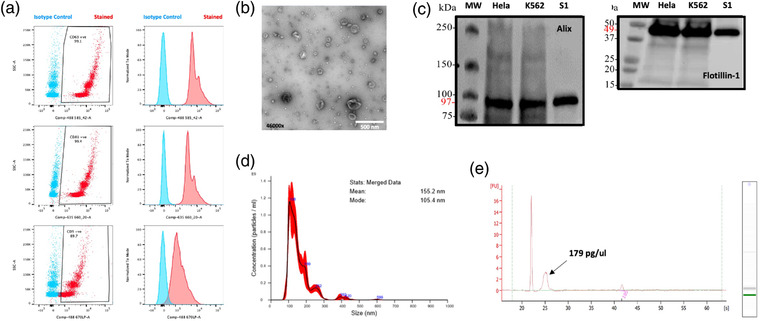
Example extracellular vesicle characterisation data. (a) Positive staining for EV markers CD63, CD81 and CD9 alongside appropriate isotype controls, assessed by flow cytometry using EVs isolated from cell culture supernatant. (b) Transmission electron microscopy showing isolated serum EVs of the expected size range (30–200 nm) and morphology (cup‐shaped vesicles). (c) Western Blot analysis of positive EV markers Flotillin‐1 and Alix expression,. (d) Nanoparticle tracking analysis based on the NanoSight, indicating EV size distribution within the expected range (mode 105.4 nm). (e) Assessment of RNA concentration isolated from EV, using the Agilent Bioanalyzer (179 pg/ul).

Extracellular vesicle characterisation was performed according to MISEV guidelines (Théry et al., 2018) using recognised methods and example data is shown for a sample taken from Dataset D. This sample was CD63 (99%), CD81 (99%) and CD9 (90%) positive by flow cytometric assessment (Figure 1a) and demonstrated typical cup‐shaped vesicular morphology by transmission electron microscopy (Figure 1b). Extracellular vesicles were positive for Alix and Flotillin‐1 according to western blot (Figure 1c) and had a modal vesicle size of 105.4 nm (+/−5.0 nm) by nanoparticle tracking analysis (Figure 1d). The extracted RNA from all samples was quantified by bioanalyser and for the example shown in Figure 1(e), the concentration was 179 pg/ul, with a size peak at the small RNA range (mean 118 nt).

### 3.3 NanoString imaging quality control

The imaging quality control (QC) is a measure of the percentage of requested fields of view (FOV) successfully scanned in each cartridge lane. The nCounter system images FOV separately and sums the barcode counts of all FOVs from a single lane to form the final raw data counts for each unique barcode. The number of FOVs successfully imaged is then reported as FOV counted. Successful imaging QC requires a minimum of 75% of the requested FOV to be successfully scanned. Our cartridges were scanned with 555FOV requested and attained the following; Data A average 513FOV (92%) (range 493–534 (89%–96%)), Data B average 543FOV (98%) (range 522–551 (94%–99%)), Data C average 546FOV (98%) (range 537–553 (97‐99%)), Data D average 539FOV (97%) (range 518–546 (93%–98%)).

### 3.4 Binding density

**FIGURE 2:**
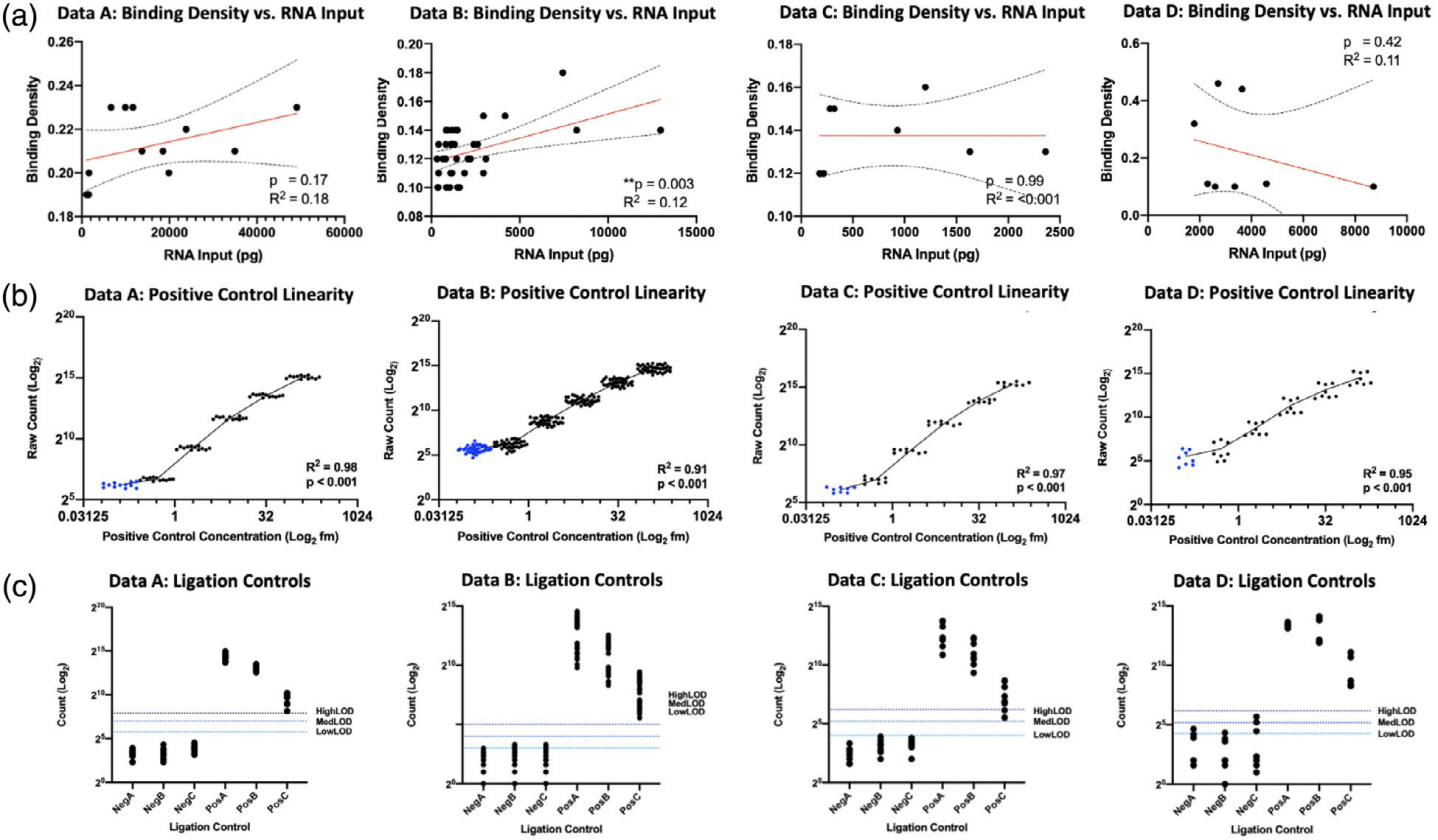
Binding density, positive control and ligation control data. (a) Correlation analysis between binding density and RNA input concentration (pg). Pearson correlation with linear regression analysis was performed for each dataset. Mean linear regression is plotted (red line) with 95% confidence intervals (dashed line). (b) Correlation analysis between raw counts (Log_2_) and positive control concentration (Log_2_ fm). Linear regression was analysed and goodness of fit R^2^ was calculated for each data set. POS_F (0.125 fm) is denoted in blue. (c) Digital counts (Log2) are shown for all ligation controls. Average LowLOD, MedLOD and HighLOD thresholds calculated for all negative controls are highlighted in shades of blue. Data A = Serum EVs isolated by precipitation reagent. Data B = Serum EVs isolated by precipitation reagent. Data C = Primary cell culture supernatant EVs isolated by precipitation reagent. Data D = Primary cell culture supernatant EVs isolated by ultracentrifugation.

The reporter probe binding density passed recommended QC parameters of 0.1–2.25 range for all samples, with average binding density of Data A = 0.21 (range 0.19–0.23) (Table S1), Data B = 0.12 (range 0.1–0.18) (Table S2), Data C = 0.14 (range 0.12–0.16) (Table S3) and Data D = 0.23 (range 0.1–0.46) (Table S4). The binding density can be a useful parameter to take into account when dealing with challenging samples, as it can act as a surrogate metric for RNA input and experimental optimisation for sample input. We observed correlation between the input RNA loaded and the binding density for Data B (Pearson R^2^ = 012, *p* = 0.003), but not Data A, C or D (Data A: Pearson R^2^ = 018, *p* = 0.17; Data C: Pearson R^2^ < 0.001, *p* = 0.99; Data D: Pearson R^2^ = 0.11, *p* = 0.42) (Figure 2a).

### 3.5 Negative controls

Every nCounter microRNA assay contains eight negative control probes, commonly used to set background thresholds. It is possible to use the mean, mean plus standard deviation, median, geometric mean, or maximum of the negative control counts. The level of stringency used to define this threshold will affect the balance between false positive and false negatives. In this study, we defined three levels of stringency for defining background and therefore, limit of detection (LOD): Low LOD = average of negative controls; Medium LOD = average of negative controls + 2*S.D; High LOD = 2*(average of negative controls + 2*S.D) (based on the nSolver 4.0 Analysis Software User Manual, NanoString nSolver Analysis online webinars and NanoString personal communication).

### 3.6 Positive controls

Six synthetic control targets are included within the assay; *POS_A* to *POS_F*. They are used to assess three QC measures: overall assay efficiency, assay linearity and limit of detection (LOD). Overall assay efficiency was demonstrated for all low concentration RNA samples within this study, as determined by assay efficiency QC. The assay linearity can be assessed by the step‐wise concentrations of positive controls, with R^2^ values > 0.95 indicating successful hybridization and/or assay performance. In the present study, positive control linearity passed QC for all low concentration RNA samples: Data A R^2^ = 0.98 (range 0.98—0.99), Data B R^2^ = 0.99 (range 0.99–0.99), Data C R^2^ = 0.97 (range 0.96–0.99), Data D R^2^ = 0.99 (range 0.98–0.99) (example graphs given Figure 2b).

The positive control limit of detection (LOD) QC indicates whether the counts for the POS_E control probe and target sequence, spiked in at 0.5fM (assumed to be the systems limit of detection) are significantly above the counts of the Negative control probes. It is expected that counts for POS_E will be higher than background. In our study, the probe count for POS_E was above lowLOD, MedLOD and HighLOD for all samples in Data B and Data C. Data A POS_E was above LowLOD for all samples, and for 2/12 samples based on MedLOD, while POS_E was below all HighLOD thresholds. In Data D, POS_E was above LowLOD and MedLOD for all samples, and above HighLOD for 6/8 samples. Of note, POS_F has a known concentration of 0.125 fM, which is considered below the limit of detection of the system. It is therefore recommended that POS_F should be excluded from the positive control linearity calculation. In our study, for Data A the probe count for POS_F was above LowLOD, but below MedLOD and HighLOD for all samples. In Data B, POS_F was above the LOD at all thresholds for all samples. In Data C, POS_F was above LowLOD and MedLOD for all samples, and for 4/8 samples based on HighLOD. For Data D, POS_F was above LowLOD for all samples, above MedLOD for 6/8 samples and above HighLOD for 5/8 samples. The positive control linearity when POS_F was included in the calculation was; Data A R^2^ = 0.98 (*p* < 0.001), Data B R^2^ = 0.91 (*p* < 0.001), Data C R^2^ = 0.97 (*p* < 0.0001) and Data D R^2^ = 0.95 (*p* < 0.001).

### 3.7 Ligation controls

Each miRNA assay contains six short synthetic RNA ligation control constructs. Three of these are subjected to ligation and release a miRNA tag as a positive ligation control, while three are not subject to ligation and serve as ligation‐negative controls. Ideally, ligation‐negative controls will yield counts in the negative control range (background level) and the ligation‐positive control counts will be significantly higher, increasing from LIG_POS_C to LIG_POS_A. In the present study, for Data A all positive ligation controls were above all thresholds of LOD, with increasing positivity from Lig_Pos_C to Lig_Pos_A. All negative ligation controls were below all thresholds of LOD (Figure 2c). For Data B, all positive controls were above the LOD of detection thresholds and all negative controls were below the MedLOD and HighLOD, while 2/36 Lig_Neg_B probes and 3/36 Lig_Neg_C probes were above the LowLOD (Figure 2c). In Data set C and D, all positive ligation controls were above all LOD thresholds, and all negative controls apart from 1 were below all LOD thresholds (Data C and Data D: Lig_Neg_C was above LowLOD) (Figure 2c).

### 3.8 Background and normalisation using negative controls

As previously described, in this exploratory study we defined three experimental levels of stringency for defining background: HighLOD, MedLOD and LowLOD. Accordingly, an individual normalisation was performed based on codeset content normalisation with all targets above each LOD average threshold. MicroRNAs with normalised counts below the LowLOD, MedLOD or HighLOD were subsequently removed from the normalised data for further analysis.

**FIGURE 3:**
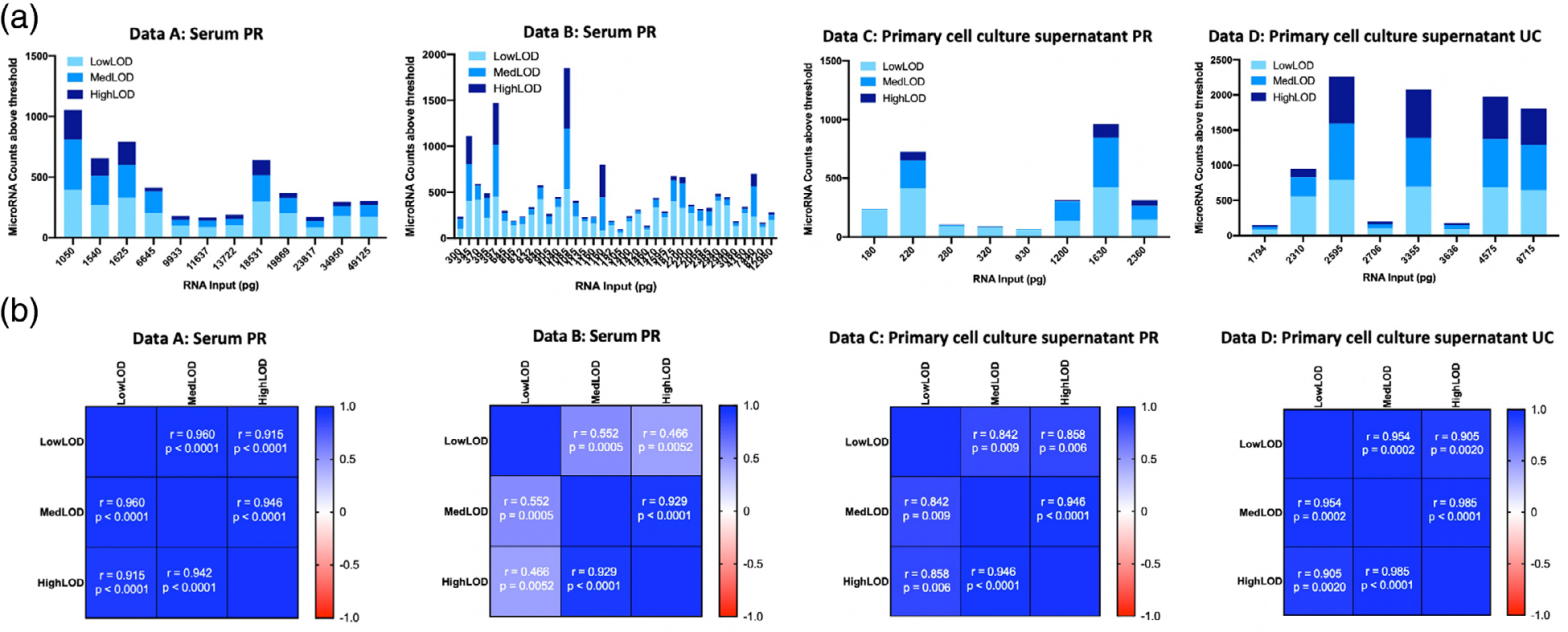
MicroRNA counts and correlation at LowLOD, MedLOD and HighLOD thresholds. (a) MicroRNA counts and correlation at LowLOD, MedLOD and HighLOD thresholds according to RNA input. The total numbers of microRNAs expressed according to HighLOD (dark blue), MedLOD (medium blue) and LowLOD (pale blue) thresholds are depicted according to the total RNA input (pg). (b) Correlation between microRNA counts at LowLOD, MedLOD and HighLOD thresholds. Pearson r‐vales and *p*‐values are shown for each correlation matrix.

For Data A (Table 1, Table S1, Figure 3a), normalisation based on LowLOD (defined as the average of negative controls) incorporated 81 targets for normalisation. The normalised data resulted in an average of 202 (range 85–393) detectable microRNAs expressed in each sample. There were 450 microRNAs of the panel that were expressed > LowLOD threshold in at least 1/12 samples, while 50 microRNAs were detectable in all 12 samples. For Data B (Table 1, Table S2, Figure 3a), LowLOD normalisation incorporated 233 targets and resulted in an average of 245 (range 64–537) microRNAs detected in each sample. There were a total of 749 microRNAs expressed above the LowLOD threshold in at least 1/36 samples, and 11 microRNAs were detected in all 36 samples. Data C (Table 1, Table S3, Figure 3a) LowLOD normalisation resulted in 148 targets for normalisation. There were an average of 199 (range 61–420) microRNAs expressed above the threshold in each sample, and 532 microRNAs of the panel expressed above LowLOD in at least 1/8 samples of the dataset, while 23 microRNA were expressed in all samples. In Data D (Table 1, Table S4, Figure 3a), normalisation based on targets above LowLOD incorporated 323 targets into the analysis, resulting in an average of 456 (range 76–798) microRNAs expressed above LowLOD in each sample. There were 798 microRNAs that were expressed in at least 1/8 samples, and 59 microRNAs that were detected in all eight samples.

**TABLE 1:** Summary of microRNA expression analysis for all data sets at each limit of detection threshold

Data set	Analysis	LowLOD	MedLOD	HighLOD
Data A	Normalisation controls	81	32	17
	MicroRNAs in > 1 sample	450	439	276
	Average microRNAs	202	153	81
	Range	85–393	50–418	22–243
	MicroRNAs in all samples	50	34	14
Data B	Normalisation controls	233	37	16
	MicroRNAs in > 1 sample	749	735	714
	Average microRNAs	245	139	72
	Range	64–537	23–657	14–657
	MicroRNAs in all samples	11	10	5
Data C	Normalisation controls	148	13	6
	MicroRNAs in > 1 sample	532	484	191
	Average microRNAs	199	126	34
	Range	61–420	6–420	2–122
	MicroRNAs in all samples	23	3	1
Data D	Normalisation controls	323	139	82
	MicroRNAs in > 1 sample	798	798	766
	Average microRNAs	456	407	335
	Range	76–798	47–798	26–692
	MicroRNAs in all samples	59	35	21

Data A = Serum EVs isolated by precipitation reagent. Data B = Serum EVs isolated by precipitation reagent. Data C = Primary cell culture supernatant EVs isolated by precipitation reagent. Data D = Primary cell culture supernatant EVs isolated by ultracentrifugation.

Based on a MedLOD threshold (average of negative controls + 2*S.D) for Data A (Table 1, Table S1, Figure 3a), 32 targets were included for normalisation. This analysis yielded an average of 153 (range 50–418) microRNAs detected in each sample. A total of 439 microRNAs expressed > MedLOD in at least 1/12 sample, while 34 microRNAs expressed across all 12 samples. Data B (Table 1, Table S2, Figure 3a), analysis was based on 37 targets above the threshold for normalisation. This resulted in 735 expressed microRNAs above MedLOD threshold, comprising an average of 139 microRNAs in each sample (range 23–657) and 10 microRNAs expressed in all 36 samples. In Data C (Table 1, Table S3, Figure 3a), a total of 13 targets were above the MedLOD threshold and used for data normalisation. This resulted in 484 microRNAs expressed > MedLOD in a minimum of 1/8 samples, with an average of 126 microRNAs in each samples (range 6–420) and three microRNAs that were expressed in all samples. For Data D (Table 1, Table S4, Figure 3a), based on MedLOD threshold, 139 targets were included for normalisation yielding an average of 407 (range 47–798) microRNAs detected in each samples. A total of 798 microRNAs were expressed > MedLOD in at least 1/8 samples, while 35 microRNAs were detected in all eight samples.

A HighLOD threshold analysis (2*(average of negative controls + 2*S.D)) for Data A (Table 1, Table S1, Figure 3a), incorporated 17 targets above threshold for normalisation. This analysis resulted in an average of 81 (range 22–243) microRNAs that were detected above HighLOD threshold in each sample. A total of 276 microRNAs were expressed > HighLOD in at least 1/12 samples, while 14 microRNAs were detected above threshold in all 12 samples. For Data B (Table 1, Table S2, Figure 3a), analysis was based on 16 targets for normalisation and resulted in expression of 714 microRNAs above HighLOD threshold, with an average of 72 microRNAs in each sample (range 14–657) and five microRNAs detected in all 36 samples. In Data C (Table 1, Table S3, Figure 3a), using a HighLOD threshold resulted in six targets for normalisation, resulting in a total of 191 microRNAs that were expressed in at least 1/8 samples with an average of 34 microRNAs expressed in each sample (range 2–122) and one microRNA that was expressed in all eight samples. In Data D (Table 1, Table S4, Figure 3a), 82 targets were above the HighLOD threshold for normalisation, yielding an average of 335 (range 26–692) microRNAs expressed in all samples. There were 766 microRNAs above HighLOD in > 1 sample, and 21 microRNAs detected in all samples.

There was no significant association detected between RNA input concentration and microRNA counts for any of the data sets analysed (Data A LowLOD *p* = 0.21, MedLOD *p* = 0.07, HighLOD *p* = 0.06; Data B LowLOD *p* = 0.45, MedLOD *p* = 0.41, HighLOD *p* = 0.46; Data C LowLOD *p* = 0.93, MedLOD *p* = 0.78, HighLOD *p* = 0.25; Data D LodLOD *p* = 0.40, MedLOD *p* = 0.29, HighLOD *p* = 0.34), indicating that the microRNA expression profiles were indicative of inherent biological variability between samples. Assessing correlation between microRNA counts for each threshold, there was significant positive correlation between LowLOD, MedLOD and HighLOD counts for all datasets (Figure 3b).

**FIGURE 4:**
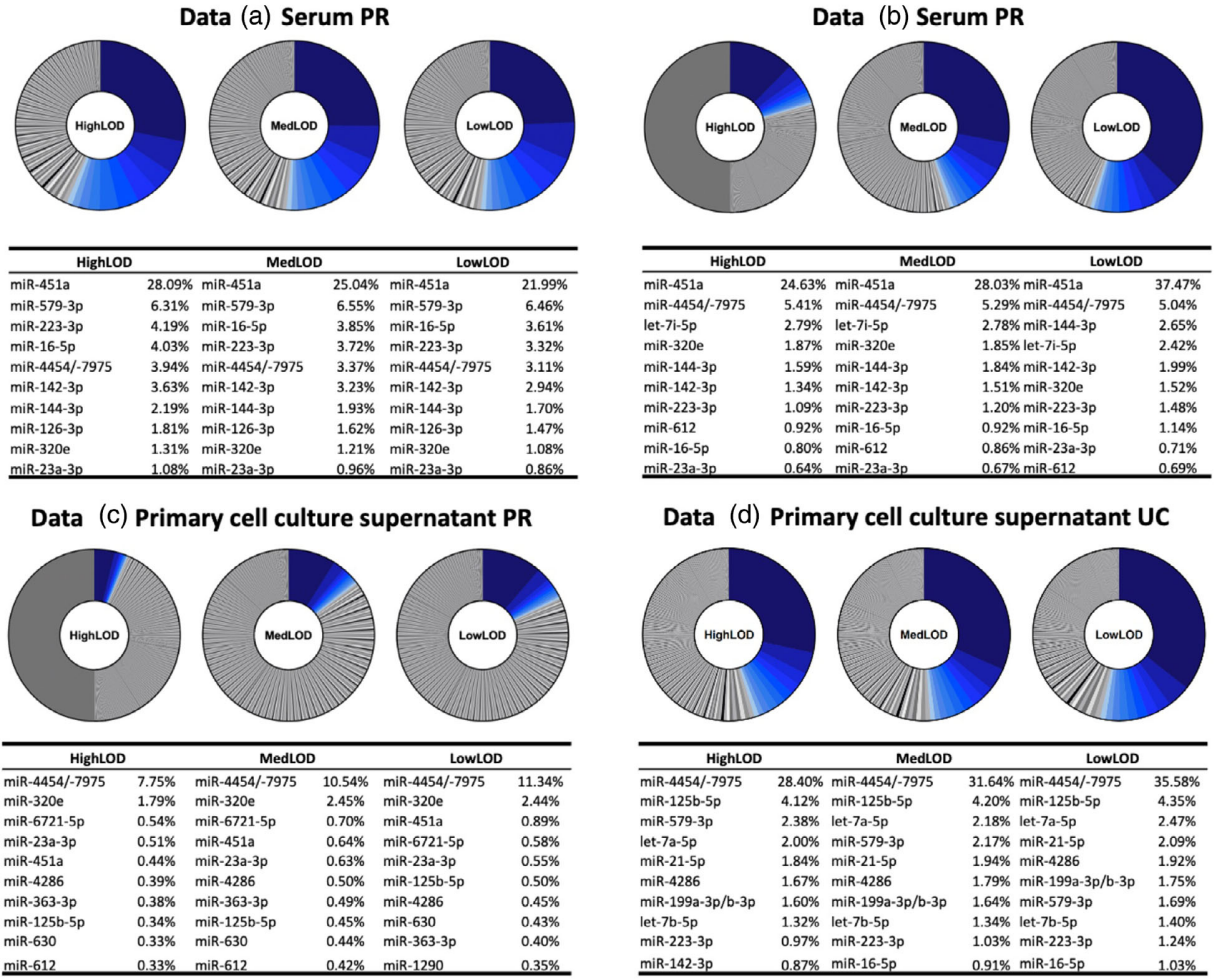
The top 10 most highly expressed microRNAs according to HighLOD, MedLOD and LowLOD. Proportional pie charts represent the percentage of microRNAs expressed for each data set at HighLOD, MedLOD and LowLOD thresholds. The top 10 most highly expressed microRNAs for each data set and LOD threshold are highlighted in shades of blue, are their % expression is specified in accompanying tables.

The top 10 most highly expressed microRNAs according to each LOD threshold showed complete overlap for Data A (Figure 4a). In Data B, there was also complete concordance with the top 10 most highly expressed microRNAs according to all LOD thresholds (Figure 4b). According to Data C, there was near complete concordance between the top 10 most highly expressed microRNAs according to all thresholds, apart from miR‐612, which was not in the top 10 highly expressed microRNAs based on LowLOD (#11) (Figure 4c). In Data D, there was complete concordance of the top 10 microRNAs, with the exception of miR‐143‐3p which was not in the top 10 microRNAs according to MedLOD (#11) and LowLOD (#11) thresholds, and miR‐16‐5p which was not in the top 10 microRNAs according to HighLOD (#12) (Figure 4d).

### 3.9 Segregation of biological sub‐groups according to MicroRNA expression

Each Data set was analysed for significant changes in microRNA expression between distinct biological sub‐groups, in order to demonstrate the ability of NanoString profiling to determine biological significance based on low RNA input. All analyses were based on MedLOD thresholds, as an intermediate level of stringency.

**FIGURE 5:**
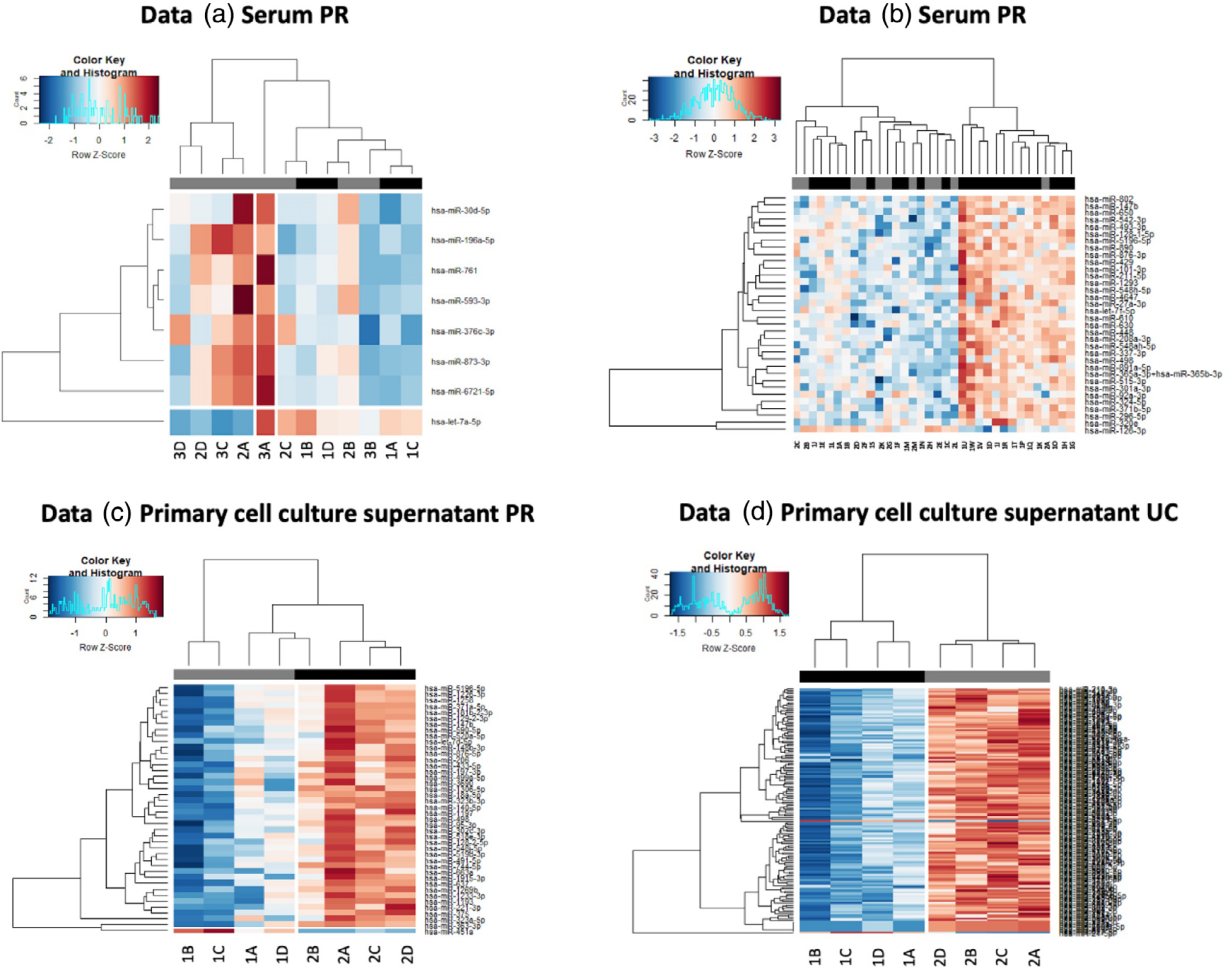
Heatmaps to show distinction of biological sub‐groups according to MicroRNA expression at MedLOD thresholds. Heatmaps for each data set are shown, detailing hierarchical clustering of significantly differentially expressed microRNAs (*p* < 0.05), between biological sub‐groups, based on normalized digital expression counts above MedLOD threshold. Each column represents an individual sample and biological sub‐groups for differential expression analysis are indicated in grey scale shading. Relative expression changes are indicated by the colour scale (red: high; blue: low). Data A: 1A‐1D = RA, 2A‐2D = OA, 3A‐3D = OIA, black scale = RA, grey scale = other arthritic conditions. Data B: 1A‐1 W = GvHD, 2A‐2 M = no GvHD, black scale = GvHD, grey scale = no GvHD. Data C: 1A‐1D = joint area 1, 2A‐2D = joint area 2, black scale = joint area 1, grey scale = joint area 2. Data D: 1A‐1D = healthy donors, 2A‐2D = OA, black scale = healthy donors, grey scale = OA.

In Data set A, there were a total of *n* = 8 microRNAs that distinguished between two populations [rheumatoid arthritis (Group 1) vs. other arthritic conditions (OA and OIA, Groups 2 and 3)] (p‐value range = < 0.001–0.049, Log_2_ FC range = −4.12–4.08). (Figure 5a). For Data set B, of note, 2/36 samples were removed from the biological analyses due to incomplete clinical notes. Within the remaining cohort of 34 samples, *n* = 34 microRNAs were significantly differently expressed between Group 1 and Group 2 samples (*p*‐value range = 0.002–0.047, Log_2_ FC range = −1.63–2.34), of which all *n* = 33 were upregulated and one microRNA was downregulated in the patients who went on to develop a haematological complication (Figure 5b). Non‐supervised hierarchical clustering analyses demonstrated distinct clustering of the biological sub‐groups, based on expression of significantly DE microRNAs (Figure 5b). In Data C, the two biological subgroups demonstrated significant DE of *n* = 42 microRNAs, of which *n* = 41 were upregulated in Group 2, while one was downregulated compared to Group 1 (*p*‐value range = 0.004–0.049, Log_2_ FC range = −4.65–5.39. The two groups showed clear separation based on unsupervised hierarchical clustering of DE microRNAs. (Figure 5c). In Data D, a signature of 141 microRNAs were significantly DE between Group 1 and Group 2 (*p*‐value range = 0.002–0.049, Log_2_ FC range = −2.78–12.05), of which two microRNAs were downregulated in Group 2, while 139 were upregulated (Figure 5d).

## 4 DISCUSSION

In a recent review evaluating the potential of circulatory microRNAs as biomarkers, 71% of studies concluded that EVs are the source of choice for miRNA biomarkers, inferring the popularity of EV biomarkersowing to advantages in quantity, quality, and stability (Nik Mohamed Kamal & Shahidan, 2020). This highlights the upmost priority to establish reproducible, accurate, sensitive, and specific platforms for EV microRNA quantification, particularly at low levels of RNA concentration, which is frequently the limitation for clinically translatable EV studies.

Common methods for global microRNA profiling include array qRT‐PCR, microarrays and small RNA sequencing (RNA‐seq). Although the use of each has been extensively reviewed and discussed elsewhere (summary Table S5), this has not always been in the context of EV microRNA, where some of the technology's individual limitations may have a greater impact. The nCounter platform aims to bridge the gap between the most common constraints of previous technologies, with the capacity to be high‐throughput, rapid turnaround, high sensitivity, and scalable to a high number of microRNAs in multiplex. However, detailed assessment of the NanoString microRNA platform for EV samples is lacking. Thus, in this study we report detailed quality control parameters and high‐level biological analysis for four EV datasets, isolated from a variety of sources and using EV isolation methods that have potential to be translated to large‐scale clinical use. We report that NanoString technology is suitable for profiling EV microRNA expression signatures based on very low input RNA concentrations, applicable to common laboratory research settings.

In this study, EV RNA concentrations as low as 180 pg were reliably assessed for microRNA expression profiles, whilst satisfying all QC parameters. This is in contrast to the general 100 ng recommendation specified in the nCounter miRNA Expression Assay User Manual, which makes the presumption that the source of RNA is cellular, where as little as 1% of the total RNA comprises small RNA (NanoString Technologies, 2019). Despite an increasing number of studies utilizing NanoString technology for EV microRNA profiling investigations (Almenar‐Pérez et al., 2020; Armstrong et al., 2018; Bhagirath et al., 2018; Bhome et al., 2017; Bracht et al., 2020; Drusco et al., 2018; Ferguson et al., 2018; Garcia‐Contreras et al., 2017; Gouin et al., 2017; Lu et al., 2018; Nakamura et al., 2015; Pillay et al., 2020; Pillay et al., 2019; Sundar et al., 2019; Xuan et al., 2019; Yeh et al., 2015; Zahoor et al., 2020) (Table S6), very few specify detailed RNA loading concentrations, nor report the QC metrics of low concentration samples. Recently, Godoy *et al* performed a comprehensive analysis of reproducibility, accuracy, sensitivity and specificity for assessing microRNA quantification in biofluids using four platforms, including cCounter (Godoy et al., 2019). When assessing microRNA expression in exRNA isolated from plasma, nCounter yielded a smaller proportion of microRNAs above background compared to other platforms and failed to detect placenta‐associated differences between pregnant and non‐pregnant serum, indicating lower sensitivity compared to sequencing‐based techniques (Godoy et al., 2019). However, it should be noted that assessments involved target concentrations for each microRNA in synthetic pools of 10 attamoles, and the equimolar synthetic pool contained approximately 12,000 RNA sequences. Thus, comparisons were based on three picomoles total at a concentration of 12 femtomoles/μl. For exRNA samples, the equivalent amount of RNA contained in 20 μl of plasma was assessed (Godoy et al., 2019). In the present study, RNA input ranged from 180 to 49,125 pg and EV RNA was isolated from 1 to 3 ml of serum, which we found to be a clinically relevant volume for research samples at our Institution. In relation to very low concentration sample RNA, which is frequently problematic for EV studies, we have also found that increasing the RNA loading volume from the recommended μl to 5 μl can assist with increasing the loading RNA concentration, whilst not compromising the hybridisation efficiency nor performance of the assay (data not shown). Interestingly, similar results were found by Veldman‐Jones, who investigated the effect of increasing the input volume of 5–15 μl, using 1 μl increments (30 μl up to 45 μl final hybridization volume). They observed no detrimental effect on expression correlation, suggesting the input volume can be increased to 10 μl, allowing a 2‐fold increase in the input RNA (Veldman‐Jones et al., 2015).

With regard to QC parameters such as FOV, binding density and ligation controls, all data sets in this study passed each parameter at each LOD threshold. Interestingly, in their evaluation of NanoString robustness and sensitivity, Veldman‐Jones et al. found that expression variability was highest for the lowest expressed genes and reduced with an increasing number of FOVs captured, however, only minimal improvement was observed using the highest setting of 555 FOV compared with 280 FOV (Veldman‐Jones et al., 2015). This suggests that FOV capture may be reduced in order to increase assay turnover, without significant detrimental effect on the variability of low expressed targets.

In order to determine false positive and negative counts, every nCounter Gene Expression assay incorporates eight negative control probes. It is possible to use the mean, mean plus standard deviation, median, geometric mean, or maximum of the negative control counts. The level of stringency used in setting this threshold will affect the balance between false positive and false negative target occurrences. The number of false positives can be minimised by setting the threshold to the mean plus two standard deviations or to the maximum of the negative control counts, however, although in this case false positives will be rare, false negatives may be relatively abundant. Conversely, a more liberal threshold such as the mean of the negative controls can be set, which will increase the number of false positives, but simultaneously decrease the number of false negatives (Technologies, 2018). In this study, we defined three levels of stringency for assessing background and therefore, limit of detection (LOD). This allowed is to explore the effect of LOD stringency on assay performance for low concentration EV RNA, allowing the user flexibility to choose an analysis pipeline stringency that is most suitable for the dataset under investigation.

Six synthetic DNA positive control targets are also incorporated into each nCounter assay, ranging in concentration (128 fM to 0.125 fM; *POS_A* to *POS_F*, respectively) to measure hybridization efficiency and check the linearity performance. In the present study, positive control linearity passed QC for all low concentration RNA samples for each dataset, based on calculations that excluded POS_F, which may be considered below the LOD. POS_E is assumed to be the systems LOD and counts for this probe were above MedLOD for all datasets, indicating this threshold to be a good balance between sensitivity and specificity for low concentration EV RNA samples. This is also in accordance with NanoString guidelines, which specify that POS_E should be detectable above the average + 2*S.D of negative controls. Interestingly, POS_F was above the MedLOD for 50/64 samples within this study, highlighting the sensitivity of the assay. For the ligation controls, again a minimum of MedLOD threshold gave the most stringent QC results for low concentration RNA samples.

In a comprehensive microRNA quality control (MiRQC) study, Mestdagh et al. assessed NanoString titration response, reflecting the platforms capacity to detect small expression changes, which in turn require high reproducibility (Mestdagh et al., 2014). Titration response was plotted and the area under the curve (AUC) metric was used as a single scale‐invariant measure. NanoString was assessed to have an AUC of 0.89, which was considerably higher than a number of qRT‐PCR approaches examined. Titration response was significantly correlated to reproducibility. We have compared technical replicates of the same sample (taken from Dataset B) and observed significant correlation in microRNA expression profile (*p* < 0.0001, R = 0.91, Figure S1). NanoString's microRNA profiling method is capable of single nucleotide mismatch discrimination, enabling assessment of differences between highly homologous microRNAs. Cross reactivity among members of the let‐7 and miR‐302 families was evaluated as a median of 7.8% in independent assessment (Mestdagh et al., 2014).

An important factor when dealing with low concentration RNA is sufficient normalisation of microRNA expression profiles, which is essential to correct for factors influencing variability in RNA quality and quantity, such as differences in initial sample concentration, RNA quality or loading as well as variability in nucleic acid recovery and possible RNA degradation (Bustin et al., 2009). This is particularly relevant when assessing RNA input concentrations at the lower limits of detection, where accurate quantification can be challenging. Sufficient normalisation generally relies of the identification of suitable endogenous control genes, which is an area still in its infancy within the EV field. Indeed, many of the previously identified reference genes are not present in EVs, and identifying reference genes in multiple cell derived EVs such as those isolated from bodily fluids is more complex than for EVs derived from a single cell type. Furthermore, multiple reference genes are generally required (Gouin et al., 2017). In this regard, the NanoString microRNA codeset includes a comprehensive repertoire of controls, including normalisation controls, allowing for a range of normalisation strategies. To adjust for differences in analyte abundance and/or quality across samples, the Content Normalization factor is calculated using reference genes. This serves to remove input variance and account for different degradation states of RNA samples (NanoString Technologies, 2018).

Interestingly, there was complete correlation between microRNA counts at each threshold of LOD, and little variation in the top 10 most highly expressed microRNAs for each dataset at each LOD. The highest correlation in counts was observed between more stringent analyses approaches (MedLOD and HighLOD). For subsequent biological analyses we based our threshold on MedLOD, which is recommended by NanoString for microRNA codesets as an acceptable balance between sensitivity and specificity. For projects where low expressing targets are common, or when the presence or absence of a transcript has an important research implication, it may be useful to use a more stringent approach in order to precisely delineate false positives.

Using MedLOD, biological analyses of each data set demonstrated informative distinction of biological subgroups, based on their microRNA expression profiles. For Data set A, there were eight microRNAs that were significantly differentially expressed between rheumatoid arthritis (Group 1) and other arthritic conditions (OA and OIA, Groups 2 and 3), indicating microRNAs that may show potential for differentiating RA from other arthritis. All patients were naïve to immune modulating therapy, indicating that clustering maps to diagnosis and not background treatments in this case. Interestingly, of these let‐7a‐5p was upregulated in RA and has previously been shown to act as a predictive factor for poorer clinical response to TNF‐inhibitors and promote inflammatory response and synovium growth in RA (Liu et al., 2019). Of the microRNAs upregulated in inflammation, several have been previously associated with inflammatory pathways (Huang et al., 2019), while miR‐30d‐5p has been previously linked with systemic inflammatory response syndrome (Caserta et al., 2018) and both direct and indirect control of inflammation (Zhang et al., 2019). For Data set B, 34 microRNAs were significantly differently expressed between Group 1 and Group 2 , effectively differentiating between patients who developed a post haematological stem cell transplant complication called graft‐versus‐host disease (GvHD), compared to those who remained disease‐free. This demonstrated the ability of NanoString EV profiling to identify potential biomarkers in this setting and interestingly, of the 34 microRNAs, several have been previously associated with the development of GvHD (Crossland et al., 2017; Wu et al., 2015; Wu et al., 2018). In Data C, the two biological subgroups represented co‐cultures of MSCs with chondrocytes from either the superficial (SZ) or the middle deep zone (MDZ) of knee articular cartilage. According to unsupervised hierarchical clustering by microRNA expression, patient samples clustered separately following the cartilage zones. There were 42 microRNAs differentially expressed between the two regions, including chondrocyte‐associated microRNAs miR‐140 (Nakamura et al., 2012; Papaioannou et al., 2013; Yang et al., 2011) miR‐18a (Ohgawara et al., 2009) and miR‐375 (Song et al., 2013). These results indicate that the miRNA profiling could distinguish between experimental conditions. Finally, in Data D, a signature of 141 microRNAs were significantly DE between Group 1 and Group 2, representing MSC‐derived EVs from osteoarthritic or healthy donors. Again, several microRNAs previously associated with osteoarthritis or MSC function were represented within this signature, further demonstrating the biological relevance of the analyses (D'adamo et al., 2016; Iliopoulos et al., 2008; Wang et al., 2016; Yang et al., 2016).

Although the present study demonstrates the utility of nCounter technology for assessment of low concentration EV RNA samples, regardless of EV isolation method tested, the effect of EV isolation methods on microRNA detection is also an important consideration. EVs isolated using different methods from the same sample are likely to yield disparate results, given the inaccuracy of many methods in isolating homogeneous populations of EV subtypes. It would therefore be of great interest to perform an in‐depth comparison of EV microRNA profiles using NanoString on the same cohort of samples, generated using a variety of EV isolation methods, and this should be the focus of further investigation.

In conclusion, NanoString technology offers a sensitive approach to the detection and profiling of EV‐derived microRNA, that may provide a solution for research studies that focus on samples with limited RNA available. We have shown that this approach can reliably be applied to RNA concentrations > 100 times lower than the manufacturer recommended starting concentration, whereby data passes rigorous QC parameters. These results may provide added confidence in the application of NanoString technology when planning EV microRNA research studies.

## AUTHOR CONTRIBUTIONS

Rachel Crossland: Conceptualization; Data curation; Formal analysis; Funding acquisition; Investigation; Methodology; Project administration; Resources; Supervision; Writing—original draft; Writing—review & editing. Anna Albiero: Data curation; Formal analysis; Investigation; Resources; Validation; Writing—review & editing. Clara Sanjurjo: Data curation; Formal analysis; Resources; Validation; Writing—review & editing. Monica Reis: Data curation; Formal analysis; Investigation; Resources; Validation; Writing—review & editing. Anastasia Resteu: Data curation; Formal analysis; Investigation; Resources; Validation; Writing—review & editing. Amy E. Anderson: Data curation; Investigation; Methodology; Resources; Validation; Writing—review & editing. Anne M. Dickinson: Funding acquisition; Project administration; Supervision; Writing—review & editing. Arthur G. Pratt: Data curation; Investigation; Project administration; Resources; Supervision; Writing—review & editing. Mark Birch: Data curation; Investigation; Resources; Supervision; Writing—review & editing. Andrew W. McCaskie: Data curation; Investigation; Resources; Supervision; Writing—review & editing. Elena Jones: Data curation; Investigation; Project administration; Resources; Supervision; Writing—review & editing. Xiao‐nong Wang: Conceptualization; Data curation; Funding acquisition; Investigation; Project administration; Resources; Supervision; Validation; Writing—original draft; Writing—review & editing

## DISCLOSURE

We acknowledge that no financial interest or benefit that has arisen from the direct applications of this research.

## CONFLICT OF INTEREST

The authors declare no conflicts of interest.

## Supporting information

Supporting Information

Supporting Information
