# Supplementary figures and images for: MicroRNA profiling of low concentration extracellular vesicle RNA utilizing NanoString nCounter technology
Source: J Extracell Biol. 2023 Jan 28;2(1):e72. doi: 10.1002/jex2.72 (PMC11080777; doi:10.1002/jex2.72)

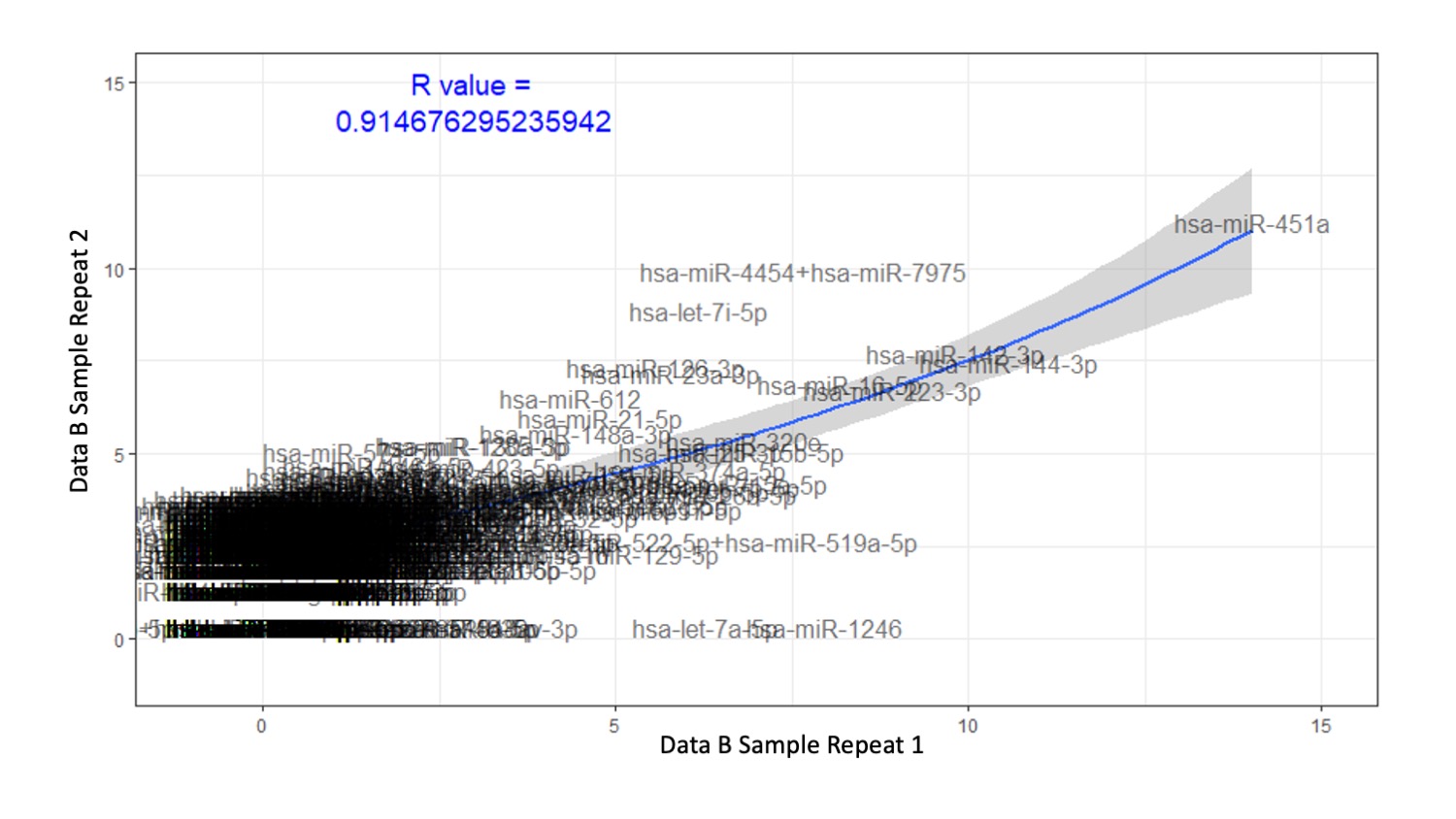

Supplement: Supplementary file 1 — Supporting Information [file JEX2-2-e72-s002.jpg]
