# Supplementary material for: MicroRNA profiling of low concentration extracellular vesicle RNA utilizing NanoString nCounter technology
Source: J Extracell Biol. 2023 Jan 28;2(1):e72. doi: 10.1002/jex2.72 (PMC11080777; doi:10.1002/jex2.72)
Supplement: Supplementary file 2 — Supporting Information [file JEX2-2-e72-s001.pptx]

## Slide 1
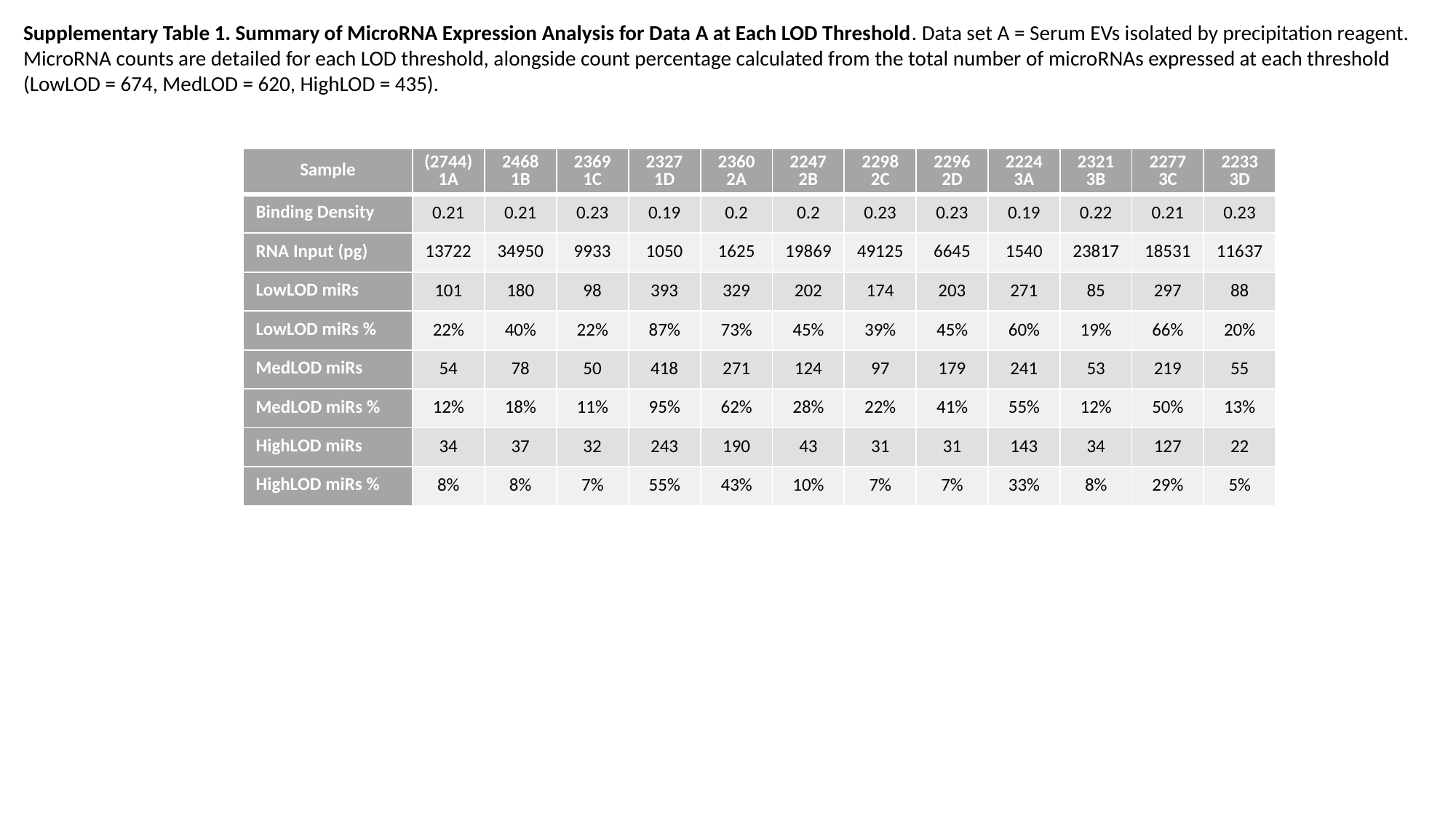

Supplementary Table 1. Summary of MicroRNA Expression Analysis for Data A at Each LOD Threshold. Data set A = Serum EVs isolated by precipitation reagent. MicroRNA counts are detailed for each LOD threshold, alongside count percentage calculated from the total number of microRNAs expressed at each threshold (LowLOD = 674, MedLOD = 620, HighLOD = 435).
| Sample | (2744) 1A | 2468 1B | 2369 1C | 2327 1D | 2360 2A | 2247 2B | 2298 2C | 2296 2D | 2224 3A | 2321 3B | 2277 3C | 2233 3D |
| --- | --- | --- | --- | --- | --- | --- | --- | --- | --- | --- | --- | --- |
| Binding Density | 0.21 | 0.21 | 0.23 | 0.19 | 0.2 | 0.2 | 0.23 | 0.23 | 0.19 | 0.22 | 0.21 | 0.23 |
| RNA Input (pg) | 13722 | 34950 | 9933 | 1050 | 1625 | 19869 | 49125 | 6645 | 1540 | 23817 | 18531 | 11637 |
| LowLOD miRs | 101 | 180 | 98 | 393 | 329 | 202 | 174 | 203 | 271 | 85 | 297 | 88 |
| LowLOD miRs % | 22% | 40% | 22% | 87% | 73% | 45% | 39% | 45% | 60% | 19% | 66% | 20% |
| MedLOD miRs | 54 | 78 | 50 | 418 | 271 | 124 | 97 | 179 | 241 | 53 | 219 | 55 |
| MedLOD miRs % | 12% | 18% | 11% | 95% | 62% | 28% | 22% | 41% | 55% | 12% | 50% | 13% |
| HighLOD miRs | 34 | 37 | 32 | 243 | 190 | 43 | 31 | 31 | 143 | 34 | 127 | 22 |
| HighLOD miRs % | 8% | 8% | 7% | 55% | 43% | 10% | 7% | 7% | 33% | 8% | 29% | 5% |

## Slide 2
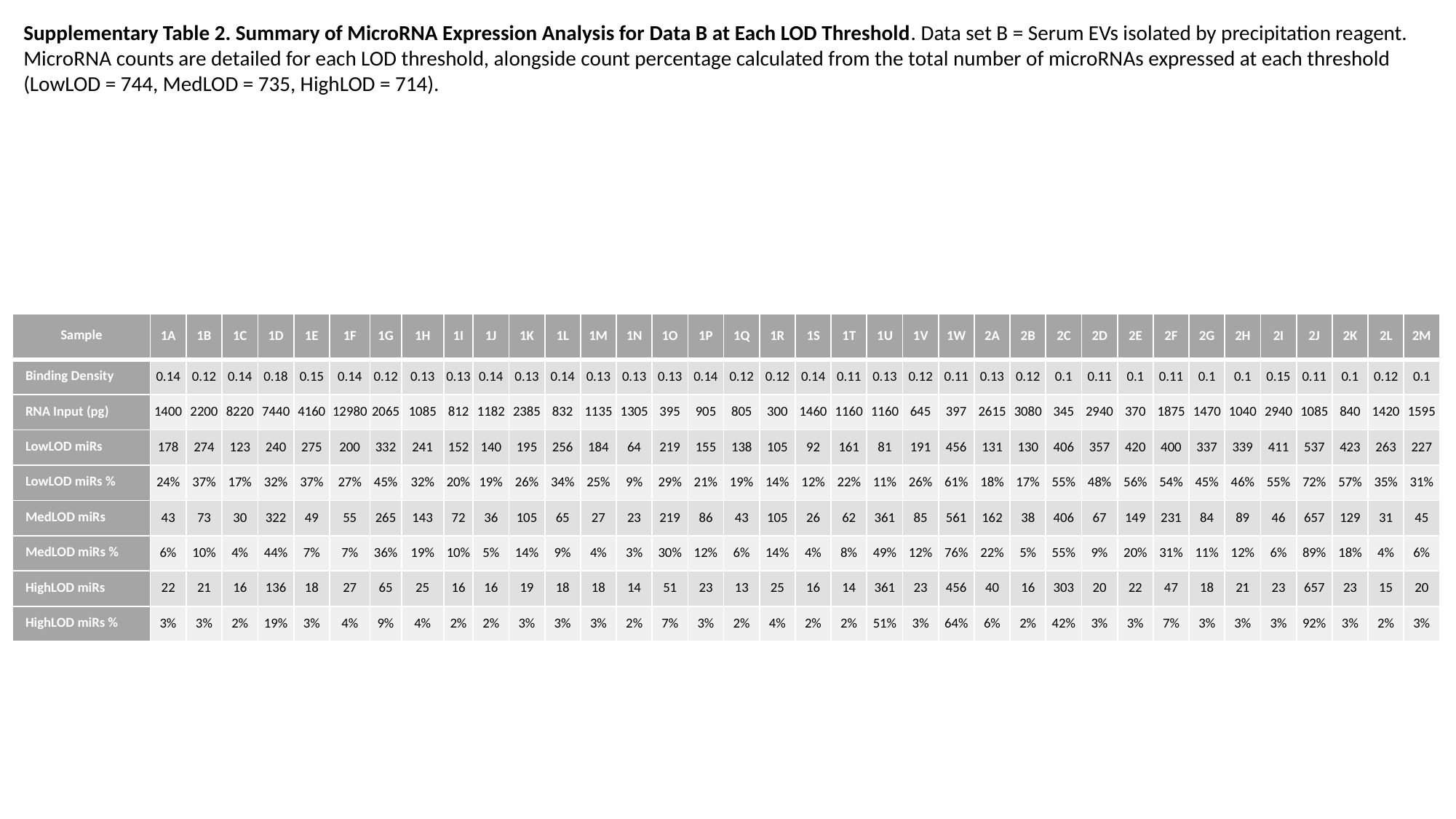

Supplementary Table 2. Summary of MicroRNA Expression Analysis for Data B at Each LOD Threshold. Data set B = Serum EVs isolated by precipitation reagent. MicroRNA counts are detailed for each LOD threshold, alongside count percentage calculated from the total number of microRNAs expressed at each threshold (LowLOD = 744, MedLOD = 735, HighLOD = 714).
| Sample | 1A | 1B | 1C | 1D | 1E | 1F | 1G | 1H | 1I | 1J | 1K | 1L | 1M | 1N | 1O | 1P | 1Q | 1R | 1S | 1T | 1U | 1V | 1W | 2A | 2B | 2C | 2D | 2E | 2F | 2G | 2H | 2I | 2J | 2K | 2L | 2M |
| --- | --- | --- | --- | --- | --- | --- | --- | --- | --- | --- | --- | --- | --- | --- | --- | --- | --- | --- | --- | --- | --- | --- | --- | --- | --- | --- | --- | --- | --- | --- | --- | --- | --- | --- | --- | --- |
| Binding Density | 0.14 | 0.12 | 0.14 | 0.18 | 0.15 | 0.14 | 0.12 | 0.13 | 0.13 | 0.14 | 0.13 | 0.14 | 0.13 | 0.13 | 0.13 | 0.14 | 0.12 | 0.12 | 0.14 | 0.11 | 0.13 | 0.12 | 0.11 | 0.13 | 0.12 | 0.1 | 0.11 | 0.1 | 0.11 | 0.1 | 0.1 | 0.15 | 0.11 | 0.1 | 0.12 | 0.1 |
| RNA Input (pg) | 1400 | 2200 | 8220 | 7440 | 4160 | 12980 | 2065 | 1085 | 812 | 1182 | 2385 | 832 | 1135 | 1305 | 395 | 905 | 805 | 300 | 1460 | 1160 | 1160 | 645 | 397 | 2615 | 3080 | 345 | 2940 | 370 | 1875 | 1470 | 1040 | 2940 | 1085 | 840 | 1420 | 1595 |
| LowLOD miRs | 178 | 274 | 123 | 240 | 275 | 200 | 332 | 241 | 152 | 140 | 195 | 256 | 184 | 64 | 219 | 155 | 138 | 105 | 92 | 161 | 81 | 191 | 456 | 131 | 130 | 406 | 357 | 420 | 400 | 337 | 339 | 411 | 537 | 423 | 263 | 227 |
| LowLOD miRs % | 24% | 37% | 17% | 32% | 37% | 27% | 45% | 32% | 20% | 19% | 26% | 34% | 25% | 9% | 29% | 21% | 19% | 14% | 12% | 22% | 11% | 26% | 61% | 18% | 17% | 55% | 48% | 56% | 54% | 45% | 46% | 55% | 72% | 57% | 35% | 31% |
| MedLOD miRs | 43 | 73 | 30 | 322 | 49 | 55 | 265 | 143 | 72 | 36 | 105 | 65 | 27 | 23 | 219 | 86 | 43 | 105 | 26 | 62 | 361 | 85 | 561 | 162 | 38 | 406 | 67 | 149 | 231 | 84 | 89 | 46 | 657 | 129 | 31 | 45 |
| MedLOD miRs % | 6% | 10% | 4% | 44% | 7% | 7% | 36% | 19% | 10% | 5% | 14% | 9% | 4% | 3% | 30% | 12% | 6% | 14% | 4% | 8% | 49% | 12% | 76% | 22% | 5% | 55% | 9% | 20% | 31% | 11% | 12% | 6% | 89% | 18% | 4% | 6% |
| HighLOD miRs | 22 | 21 | 16 | 136 | 18 | 27 | 65 | 25 | 16 | 16 | 19 | 18 | 18 | 14 | 51 | 23 | 13 | 25 | 16 | 14 | 361 | 23 | 456 | 40 | 16 | 303 | 20 | 22 | 47 | 18 | 21 | 23 | 657 | 23 | 15 | 20 |
| HighLOD miRs % | 3% | 3% | 2% | 19% | 3% | 4% | 9% | 4% | 2% | 2% | 3% | 3% | 3% | 2% | 7% | 3% | 2% | 4% | 2% | 2% | 51% | 3% | 64% | 6% | 2% | 42% | 3% | 3% | 7% | 3% | 3% | 3% | 92% | 3% | 2% | 3% |

## Slide 3
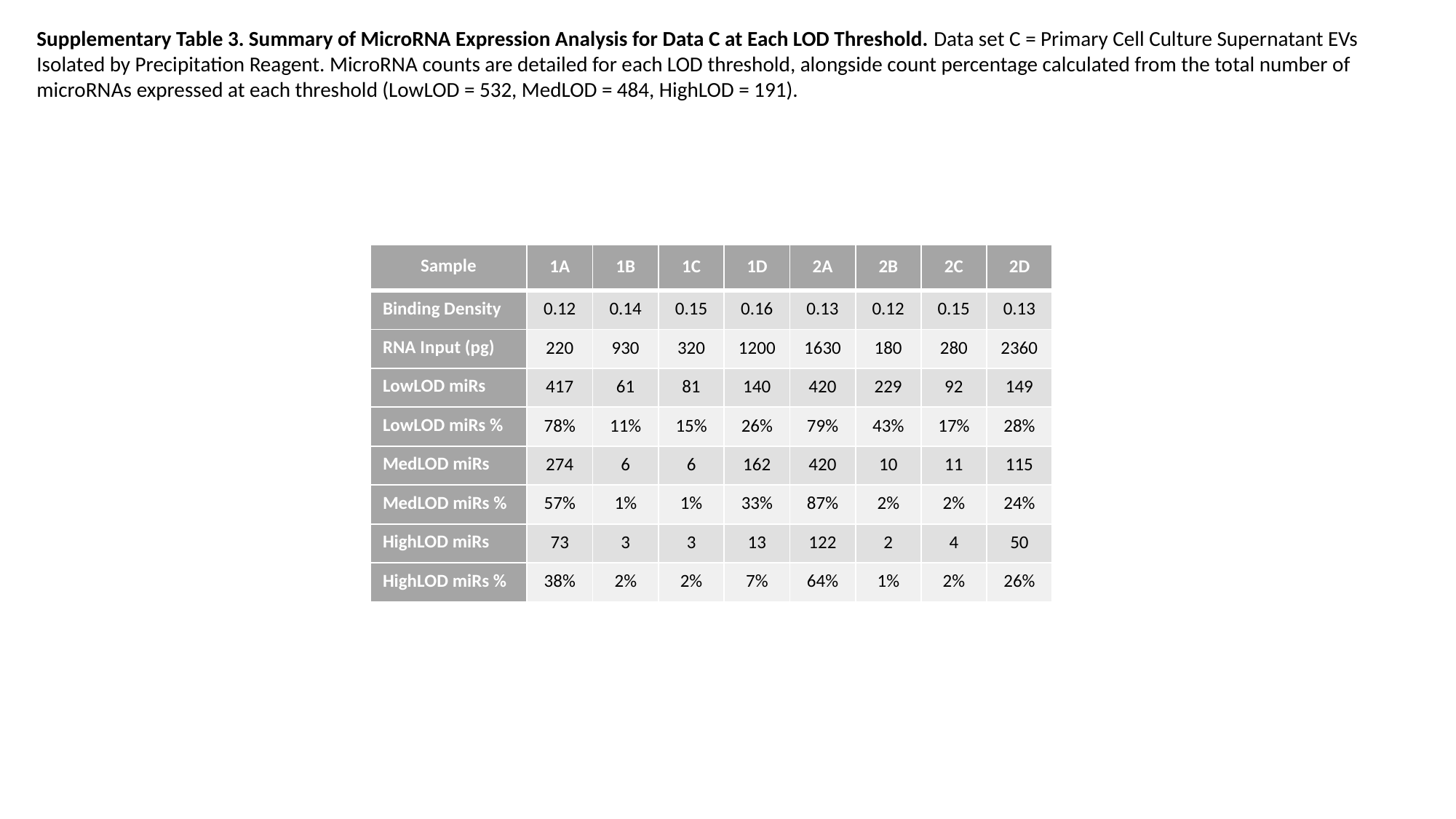

Supplementary Table 3. Summary of MicroRNA Expression Analysis for Data C at Each LOD Threshold. Data set C = Primary Cell Culture Supernatant EVs Isolated by Precipitation Reagent. MicroRNA counts are detailed for each LOD threshold, alongside count percentage calculated from the total number of microRNAs expressed at each threshold (LowLOD = 532, MedLOD = 484, HighLOD = 191).
| Sample | 1A | 1B | 1C | 1D | 2A | 2B | 2C | 2D |
| --- | --- | --- | --- | --- | --- | --- | --- | --- |
| Binding Density | 0.12 | 0.14 | 0.15 | 0.16 | 0.13 | 0.12 | 0.15 | 0.13 |
| RNA Input (pg) | 220 | 930 | 320 | 1200 | 1630 | 180 | 280 | 2360 |
| LowLOD miRs | 417 | 61 | 81 | 140 | 420 | 229 | 92 | 149 |
| LowLOD miRs % | 78% | 11% | 15% | 26% | 79% | 43% | 17% | 28% |
| MedLOD miRs | 274 | 6 | 6 | 162 | 420 | 10 | 11 | 115 |
| MedLOD miRs % | 57% | 1% | 1% | 33% | 87% | 2% | 2% | 24% |
| HighLOD miRs | 73 | 3 | 3 | 13 | 122 | 2 | 4 | 50 |
| HighLOD miRs % | 38% | 2% | 2% | 7% | 64% | 1% | 2% | 26% |

## Slide 4
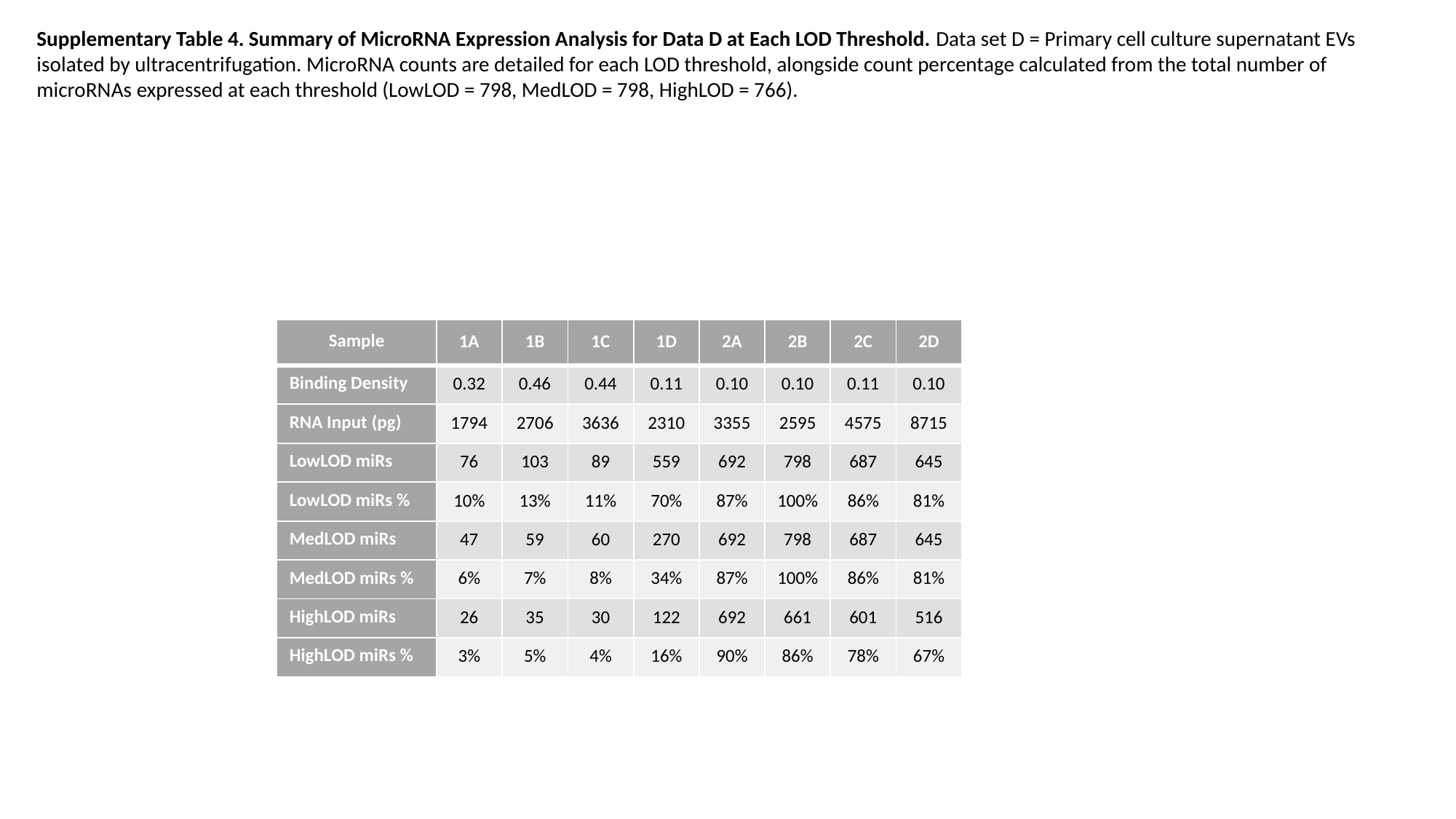

Supplementary Table 4. Summary of MicroRNA Expression Analysis for Data D at Each LOD Threshold. Data set D = Primary cell culture supernatant EVs isolated by ultracentrifugation. MicroRNA counts are detailed for each LOD threshold, alongside count percentage calculated from the total number of microRNAs expressed at each threshold (LowLOD = 798, MedLOD = 798, HighLOD = 766).
| Sample | 1A | 1B | 1C | 1D | 2A | 2B | 2C | 2D |
| --- | --- | --- | --- | --- | --- | --- | --- | --- |
| Binding Density | 0.32 | 0.46 | 0.44 | 0.11 | 0.10 | 0.10 | 0.11 | 0.10 |
| RNA Input (pg) | 1794 | 2706 | 3636 | 2310 | 3355 | 2595 | 4575 | 8715 |
| LowLOD miRs | 76 | 103 | 89 | 559 | 692 | 798 | 687 | 645 |
| LowLOD miRs % | 10% | 13% | 11% | 70% | 87% | 100% | 86% | 81% |
| MedLOD miRs | 47 | 59 | 60 | 270 | 692 | 798 | 687 | 645 |
| MedLOD miRs % | 6% | 7% | 8% | 34% | 87% | 100% | 86% | 81% |
| HighLOD miRs | 26 | 35 | 30 | 122 | 692 | 661 | 601 | 516 |
| HighLOD miRs % | 3% | 5% | 4% | 16% | 90% | 86% | 78% | 67% |

## Slide 5
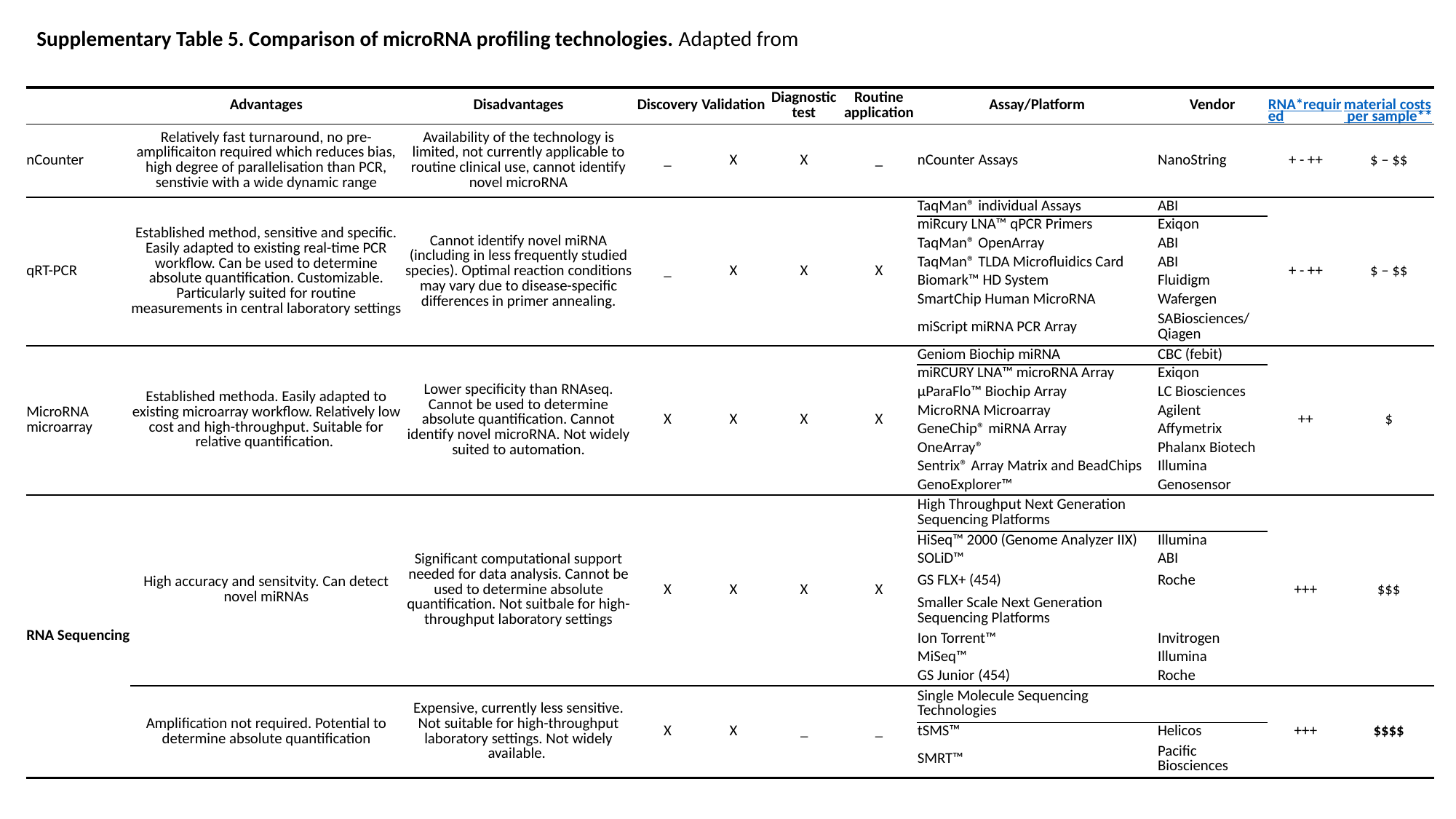

Supplementary Table 5. Comparison of microRNA profiling technologies. Adapted from
| | Advantages | Disadvantages | Discovery | Validation | Diagnostic test | Routine application | Assay/Platform | Vendor | RNA\*required | material costs per sample\*\* |
| --- | --- | --- | --- | --- | --- | --- | --- | --- | --- | --- |
| nCounter | Relatively fast turnaround, no pre-amplificaiton required which reduces bias, high degree of parallelisation than PCR, senstivie with a wide dynamic range | Availability of the technology is limited, not currently applicable to routine clinical use, cannot identify novel microRNA | \_ | X | X | \_ | nCounter Assays | NanoString | + - ++ | $ – $$ |
| qRT-PCR | Established method, sensitive and specific. Easily adapted to existing real-time PCR workflow. Can be used to determine absolute quantification. Customizable. Particularly suited for routine measurements in central laboratory settings | Cannot identify novel miRNA (including in less frequently studied species). Optimal reaction conditions may vary due to disease-specific differences in primer annealing. | \_ | X | X | X | TaqMan® individual Assays | ABI | + - ++ | $ – $$ |
| | | | | | | | miRcury LNA™ qPCR Primers | Exiqon | | |
| | | | | | | | TaqMan® OpenArray | ABI | | |
| | | | | | | | TaqMan® TLDA Microfluidics Card | ABI | | |
| | | | | | | | Biomark™ HD System | Fluidigm | | |
| | | | | | | | SmartChip Human MicroRNA | Wafergen | | |
| | | | | | | | miScript miRNA PCR Array | SABiosciences/Qiagen | | |
| MicroRNA microarray | Established methoda. Easily adapted to existing microarray workflow. Relatively low cost and high-throughput. Suitable for relative quantification. | Lower specificity than RNAseq. Cannot be used to determine absolute quantification. Cannot identify novel microRNA. Not widely suited to automation. | X | X | X | X | Geniom Biochip miRNA | CBC (febit) | ++ | $ |
| | | | | | | | miRCURY LNA™ microRNA Array | Exiqon | | |
| | | | | | | | μParaFlo™ Biochip Array | LC Biosciences | | |
| | | | | | | | MicroRNA Microarray | Agilent | | |
| | | | | | | | GeneChip® miRNA Array | Affymetrix | | |
| | | | | | | | OneArray® | Phalanx Biotech | | |
| | | | | | | | Sentrix® Array Matrix and BeadChips | Illumina | | |
| | | | | | | | GenoExplorer™ | Genosensor | | |
| RNA Sequencing | High accuracy and sensitvity. Can detect novel miRNAs | Significant computational support needed for data analysis. Cannot be used to determine absolute quantification. Not suitbale for high-throughput laboratory settings | X | X | X | X | High Throughput Next Generation Sequencing Platforms | | +++ | $$$ |
| | | | | | | | HiSeq™ 2000 (Genome Analyzer IIX) | Illumina | | |
| | | | | | | | SOLiD™ | ABI | | |
| | | | | | | | GS FLX+ (454) | Roche | | |
| | | | | | | | Smaller Scale Next Generation Sequencing Platforms | | | |
| | | | | | | | Ion Torrent™ | Invitrogen | | |
| | | | | | | | MiSeq™ | Illumina | | |
| | | | | | | | GS Junior (454) | Roche | | |
| | Amplification not required. Potential to determine absolute quantification | Expensive, currently less sensitive. Not suitable for high-throughput laboratory settings. Not widely available. | X | X | \_ | \_ | Single Molecule Sequencing Technologies | | +++ | $$$$ |
| | | | | | | | tSMS™ | Helicos | | |
| | | | | | | | SMRT™ | Pacific Biosciences | | |

## Slide 6
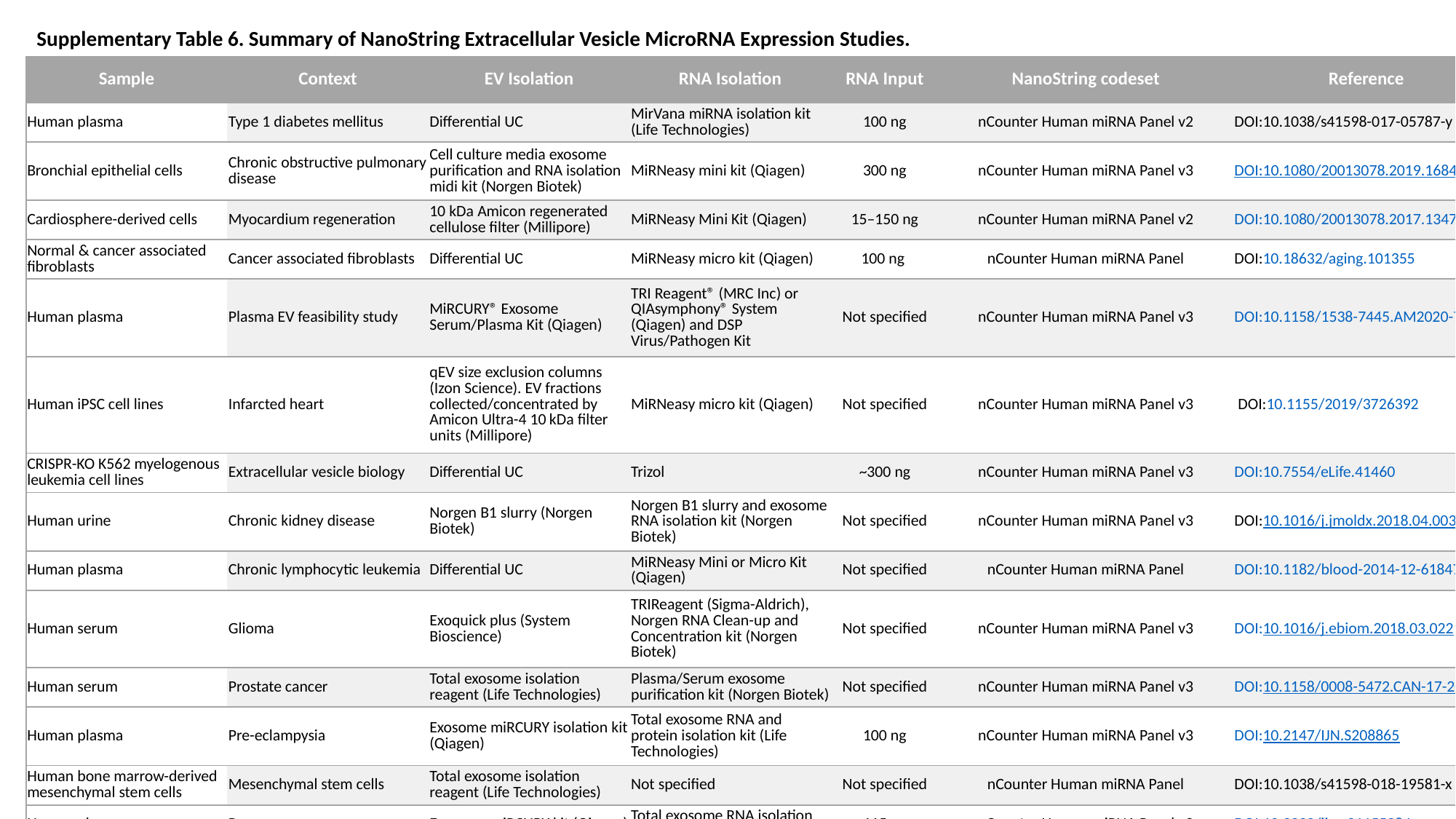

Supplementary Table 6. Summary of NanoString Extracellular Vesicle MicroRNA Expression Studies.
| Sample | Context | EV Isolation | RNA Isolation | RNA Input | NanoString codeset | Reference |
| --- | --- | --- | --- | --- | --- | --- |
| Human plasma | Type 1 diabetes mellitus | Differential UC | MirVana miRNA isolation kit (Life Technologies) | 100 ng | nCounter Human miRNA Panel v2 | DOI:10.1038/s41598-017-05787-y |
| Bronchial epithelial cells | Chronic obstructive pulmonary disease | Cell culture media exosome purification and RNA isolation midi kit (Norgen Biotek) | MiRNeasy mini kit (Qiagen) | 300 ng | nCounter Human miRNA Panel v3 | DOI:10.1080/20013078.2019.1684816 |
| Cardiosphere-derived cells | Myocardium regeneration | 10 kDa Amicon regenerated cellulose filter (Millipore) | MiRNeasy Mini Kit (Qiagen) | 15–150 ng | nCounter Human miRNA Panel v2 | DOI:10.1080/20013078.2017.1347019 |
| Normal & cancer associated fibroblasts | Cancer associated fibroblasts | Differential UC | MiRNeasy micro kit (Qiagen) | 100 ng | nCounter Human miRNA Panel | DOI:10.18632/aging.101355 |
| Human plasma | Plasma EV feasibility study | MiRCURY® Exosome Serum/Plasma Kit (Qiagen) | TRI Reagent® (MRC Inc) or QIAsymphony® System (Qiagen) and DSP Virus/Pathogen Kit | Not specified | nCounter Human miRNA Panel v3 | DOI:10.1158/1538-7445.AM2020-760 |
| Human iPSC cell lines | Infarcted heart | qEV size exclusion columns (Izon Science). EV fractions collected/concentrated by Amicon Ultra-4 10 kDa filter units (Millipore) | MiRNeasy micro kit (Qiagen) | Not specified | nCounter Human miRNA Panel v3 | DOI:10.1155/2019/3726392 |
| CRISPR-KO K562 myelogenous leukemia cell lines | Extracellular vesicle biology | Differential UC | Trizol | ~300 ng | nCounter Human miRNA Panel v3 | DOI:10.7554/eLife.41460 |
| Human urine | Chronic kidney disease | Norgen B1 slurry (Norgen Biotek) | Norgen B1 slurry and exosome RNA isolation kit (Norgen Biotek) | Not specified | nCounter Human miRNA Panel v3 | DOI:10.1016/j.jmoldx.2018.04.003 |
| Human plasma | Chronic lymphocytic leukemia | Differential UC | MiRNeasy Mini or Micro Kit (Qiagen) | Not specified | nCounter Human miRNA Panel | DOI:10.1182/blood-2014-12-618470 |
| Human serum | Glioma | Exoquick plus (System Bioscience) | TRIReagent (Sigma-Aldrich), Norgen RNA Clean-up and Concentration kit (Norgen Biotek) | Not specified | nCounter Human miRNA Panel v3 | DOI:10.1016/j.ebiom.2018.03.022 |
| Human serum | Prostate cancer | Total exosome isolation reagent (Life Technologies) | Plasma/Serum exosome purification kit (Norgen Biotek) | Not specified | nCounter Human miRNA Panel v3 | DOI:10.1158/0008-5472.CAN-17-2069 |
| Human plasma | Pre-eclampysia | Exosome miRCURY isolation kit (Qiagen) | Total exosome RNA and protein isolation kit (Life Technologies) | 100 ng | nCounter Human miRNA Panel v3 | DOI:10.2147/IJN.S208865 |
| Human bone marrow-derived mesenchymal stem cells | Mesenchymal stem cells | Total exosome isolation reagent (Life Technologies) | Not specified | Not specified | nCounter Human miRNA Panel | DOI:10.1038/s41598-018-19581-x |
| Human plasma | Pregnancy | Exosome miRCURY kit (Qiagen) | Total exosome RNA isolation kit (Life Technologies) | 115 ng | nCounter Human miRNA Panel v3 | DOI:10.3390/ijms21155384 |
| Human bone marrow-derived mesenchymal stem cells | Skeletal muscle regeneration | Differential UC | MirVana miRNA isolation kit (Life Technologies) | >5 ng | nCounter Human miRNA Panel | DOI:10.1016/j.febslet.2015.03.031 |
| Human milk | HIV-1 Infection | Total exosomes isolation reagent (from other body fluids) (Thermo Fisher) | Total exosome RNA and protein isolation kit (Invitrogen) | Not specified | nCounter Human miRNA Panel | DOI:10.1038/s41598-020-69799-x |
| Human plasma | Chronic fatigue syndrome | Total exosome isolation reagent (Life Technologies) | RNAzol (Molecular Research Center) | 100 ng | nCounter Human miRNA Panel v3 | DOI:10.1038/s41598-020-58506-5 |
